# VHL deficiency augments anthracycline sensitivity of clear cell renal cell carcinomas by down-regulating ALDH2

**DOI:** 10.1038/ncomms15337

**Published:** 2017-06-15

**Authors:** Yao-Hui Gao, Zhao-Xia Wu, Li-Qi Xie, Cai-Xia Li, Yu-Qin Mao, Yan-Tao Duan, Bing Han, San-Feng Han, Yun Yu, Hao-Jie Lu, Peng-Yuan Yang, Tian-Rui Xu, Jing-Lin Xia, Guo-Qiang Chen, Li-Shun Wang

**Affiliations:** 1 Institute of Fudan-Minhang Academic Health System, Minhang Hospital, Fudan University, 170 Xinsong Road, Shanghai 201199, China; 2 Shanghai Universities E-Institute for Chemical Biology, Key Laboratory of Cell Differentiation and Apoptosis of Chinese Ministry of Education, Ruijin Hospital, Shanghai Jiaotong University School of Medicine, 280 South Chongqing Road, Shanghai 200025, China; 3 Faculty of Life Science and Technology, Kunming University of Science and Technology, Kunming, Yunnan 650500, China

## Abstract

The von Hippel-Lindau (VHL) is deficient in ∼70% of clear-cell renal cell carcinomas (ccRCC), which contributes to the carcinogenesis and drug resistance of ccRCC. Here we show that VHL-deficient ccRCC cells present enhanced cytotoxicity of anthracyclines in a hypoxia-inducible factor-independent manner. By subtractive proteomic analysis coupling with RNAi or overexpression verification, aldehyde dehydrogenase 2 (ALDH2) is found to be transcriptionally regulated by VHL and contributes to enhanced anthracyclines cytotoxicity in ccRCC cells. Furthermore, VHL regulates ALDH2 expression by directly binding the promoter of −130 bp to −160 bp to activate the transcription of hepatocyte nuclear factor 4 alpha (HNF-4α). In addition, a positive correlation is found among the protein expressions of VHL, HNF-4α and ALDH2 in ccRCC samples. These findings will deepen our understanding of VHL function and shed light on precise treatment for ccRCC patients.

## Abstract (web summary)

The VHL tumour suppressor gene is lost in approximately 70% of clear cell renal cell carcinoma (ccRCC). In this study, the authors demonstrate that VHL loss in these tumours augments anthracyclines chemotherapy by down-regulation of ALDH2.

Renal cell carcinoma (RCC) is the most common type of kidney cancer, accounting for 2–3% of all adult tumours^1^,^2^. To date, surgery is the main treatment for this kind of cancer, while RCC is notoriously resistant to conventional chemotherapy, possibly through high expression of some multidrug resistance genes or inactivation of apoptotic pathways^3^. Also, most of these patients suffer from the side effects of chemotherapy. More recently, some new drugs targeting vascular endothelial growth factor receptor, such as sunitinib and sorafenib, have been proven to be beneficial for RCC^4^. Unfortunately, large portion of kidney cancer patients treated with these inhibitors will eventually experience disease progression. Drug treatment of kidney cancer is still unsatisfactory^5^. Therefore, it is urgent to identify the potential therapeutic targets and chemotherapeutic agents for RCC.

Clear-cell RCC (ccRCC), the most frequent and malignant type of RCC, is characterized by early loss of the von Hippel-Lindau (*VHL*) tumour-suppressor gene in most tumours^6^. The *VHL* gene encodes two isoforms, that is, a 24–30 kDa isoform with 213-amino-acid and a 19 kDa one with 160-amino-acid^3^. Early functional studies suggested that both isoforms have tumour-suppressor activity *in vivo*^6^. Functional studies indicated that VHL is an E3 ubiquitin ligase that targets the oxygen-sensitive α subunit of the hypoxia-inducible factor (HIF-α) for proteasomal degradation. When oxygen is available, HIF-α is hydroxylated on two proline residues by the oxygen-dependent HIF-α-specific prolyl hydroxylases. The hydroxylated HIF-α is ubiquitinated by VHL, followed by proteasomal degradation^4^,^7^. Under hypoxic conditions, HIF-α is stabilized due to the inhibition of hydroxylation and ubiquitination, and it subsequently translocates into the nucleus where it forms heterodimer with constitutively expressed HIF-1β (refs 7, 8). The HIF-α/HIF-1β heterodimer binds to hypoxia-responsive elements (HREs) in the promoters and activates the transcription of its targeted genes, which are involved in energy metabolism, angiogenesis, cell proliferation/differentiation and invasion/metastasis^9^,^10^,^11^,^12^,^13^. In addition to its role in HIF regulation, VHL is also implicated in a variety of HIF-independent processes, including regulation of the extracellular matrix, microtubule stabilization and maintenance of the primary cilium, control of cell senescence, and modulation of RNA polymerase II subunits^14^,^15^,^16^,^17^,^18^. However, whether VHL is a potential therapeutic target for ccRCC is currently unknown.

Aldehyde dehydrogenase belongs to a family of oxidizing enzymes that is responsible for the detoxification of aldehydes^19^,^20^. The mitochondrial aldehyde dehydrogenase 2 (ALDH2) is well known for its critical role in metabolism of acetaldehyde. In particular, ALDH2 plays a key role in oxidizing endogenous aldehydic products, such as 4-hydroxy-2-nonenal (4-HNE) and malondialdehyde, which arise from lipid peroxidation under oxidative stress^19^,^20^. ALDH2, which is expressed ubiquitously but abundant in liver, kidney, brain and heart^21^,^22^, has been regarded as a crucial enzyme in protecting the heart from oxidative stress^23^. ALDH2 dysfunction may contribute to a variety of human diseases such as cardiovascular diseases, neurodegenerative diseases, diabetes, stroke and cancer^19^,^24^,^25^,^26^,^27^.

Anthracyclines, mainly including daunorubicin, doxorubicin and epirubicin, are still widely used in modern cancer treatments despite the advent of targeted therapy. In general, anthracyclines are applied to first- or second-line standard chemotherapy in treating childhood leukaemia, lymphomas and solid malignancies by intravenous administration to induce cancer-cell growth inhibition^28^,^29^,^30^.

Here we report that VHL-deficiency augments ccRCC sensitivity to anthracyclines by downregulation of ALDH2 in an E3 ubiquitin ligase-independent manner.

## Results

### VHL-deficiency augments cytotoxicity of anthracyclines

**Figure 1:**
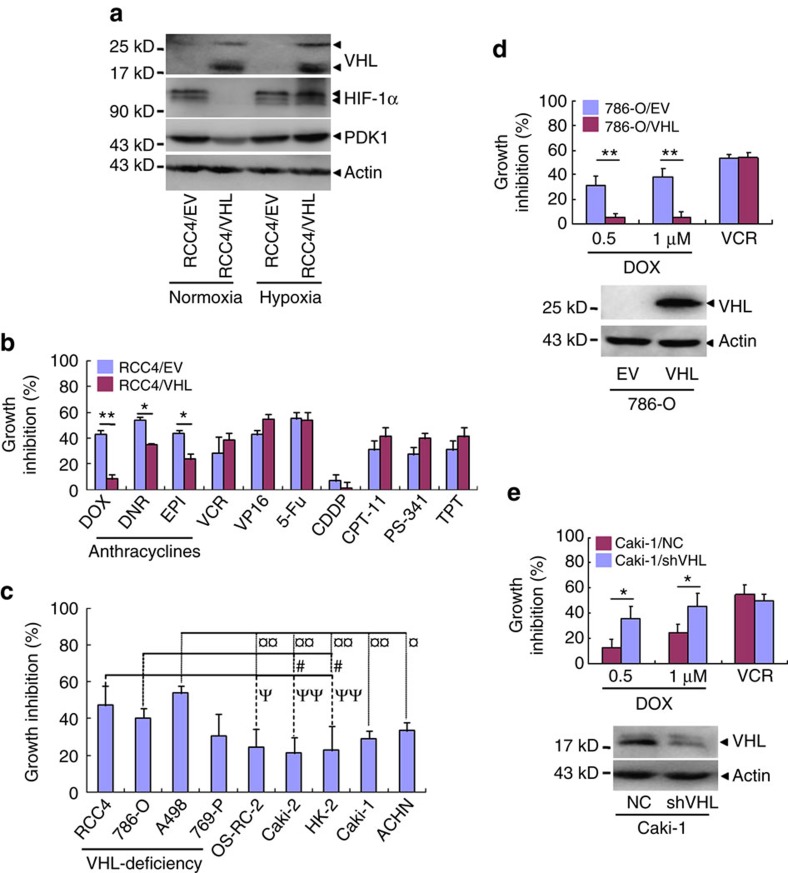
VHL-deficiency augments the cytotoxicity of anthracyclines in ccRCC. (**a**) RCC4/EV and RCC4/VHL were incubated in normoxia or hypoxia for 24 h and the indicated proteins expression was detected by western blot. (**b**) Different chemotherapeutic drugs were applied to treat RCC4/EV and RCC4/VHL cells for 24 h and cell growth inhibition rates were detected by CCK-8. (**c**) Doxorubicin was used to treat different ccRCC cell lines for 24 h and cell growth inhibition rates were detected and analysed using one-way ANOVA test followed by Bonferroni correction for *post hoc* test. ψ, # and 
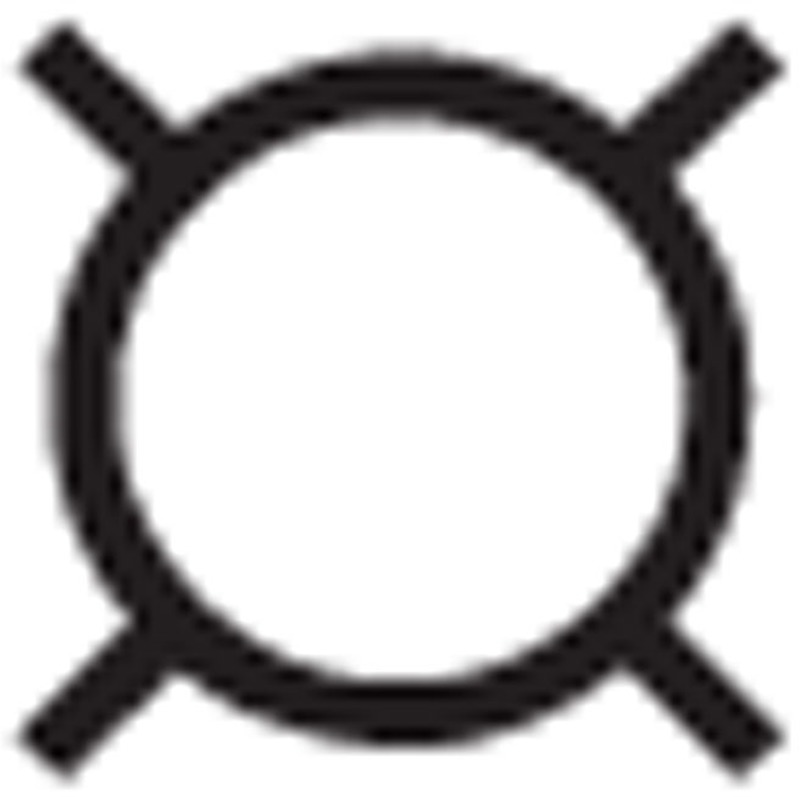
, respectively, represent the difference of RCC4, 786-O and A498 versus OS-RC-2, Caki-2 , HK-2, Caki-1 or ACHN. (ψ, # and 
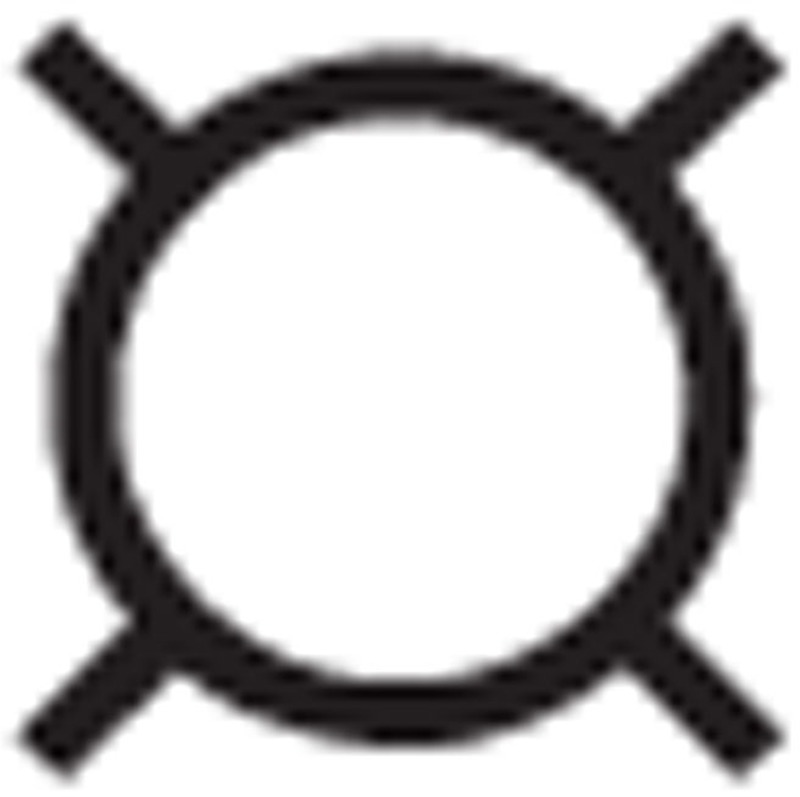
, *P*<0.05, ψψ and 
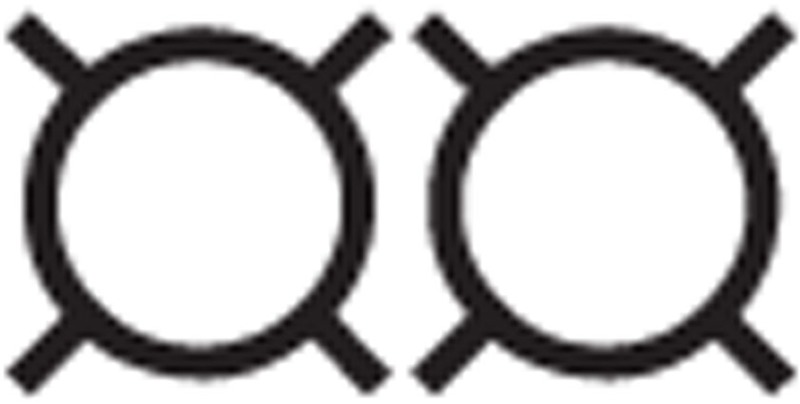
, *P*<0.01). (**d**,**e**) Western blot for VHL with actin as a loading control (bottom) and cell growth inhibition rates after treatment with doxorubicin or 1 μM VCR for 24 h (Top). (**d**) 786-O cells were stably transfected with VHL expression vector (VHL) or EV. (**e**) Caki-1 cells were infected with retroviral vectors harbouring shRNAs against VHL (shVHL) or NC. The column represents mean with bar as s.d. of three independent experiments with triplicate samples. (*P<0.05,**P<0.01 for *t*-test). ANOVA, analysis of variance; EPI, epirubicin; EV, empty vector; CDDP, cis-Diaminedichloroplatinum; CPT-11, irinotecan; DOX, doxorubicin and; DNR, daunomycin; 5-Fu, fluorouracil; NC, non-specific control; PS-341, bortezomib; TPT, topotecan; VCR, vincristine; VP-16, vepeside.

RCC4, a VHL-defective ccRCC cell line, was stably transfected with empty vector (RCC4/EV) or *VHL* (RCC4/VHL). As shown in Fig. 1a, RCC4/EV, but not RCC4/VHL cells, expressed higher levels of HIF-1α and pyruvate dehydrogenase kinase 1 (*PDK1*, a HIF-1α-responsive gene^9^) under normoxia, while HIF-1α and PDK1 proteins were increased in RCC4/VHL cells under hypoxia (1% O_2_). We compared the growth inhibition of RCC4/EV and RCC4/VHL under chemotherapeutic drugs. The results demonstrated that all three anthracycline drugs, but not non-anthracycline chemotherapeutic drugs tested, had higher growth inhibition rates to RCC4/EV than RCC4/VHL cells (Fig. 1b). Also, the cell growth inhibitory ability of doxorubicin at three different concentrations was always higher in RCC4/EV than in RCC4/VHL cells (Supplementary Fig. 1a). The half maximal inhibitory concentrations (IC50) of all three anthracyclines tested in RCC4/VHL were over 2.9 folds more than those in RCC4/EV (Supplementary Fig. 1b).

To consolidate the potential role of VHL in cell sensitivity to anthracyclines, doxorubicin, with vincristine as a control, was applied to a series of ccRCC cell lines, including VHL-deficient RCC4, 786-O, A498 and 769-P, as well as VHL-proficient Caki-1, Caki-2, OS-RC-2, HK-2 and ACHN^31^. The results revealed that the VHL-deficient ccRCC cells except for 769-P demonstrated a higher growth inhibitory response to doxorubicin than VHL-proficient ccRCC cells tested (Fig. 1c, Supplementary Fig. 1c). Similar results were shown in the comparison of VHL-deficient 786-O/EV cells with their counterpart 786-O/VHL (Fig. 1d). In consistence with this, VHL-proficient Caki-1 cells that stably expressed shRNA specifically against VHL (Caki-1/shVHL) presented higher sensitivity to doxorubicin compared with their counterpart with transfection of non-specific shRNA (Caki-1/NC, Fig. 1e).

**Figure 2:**
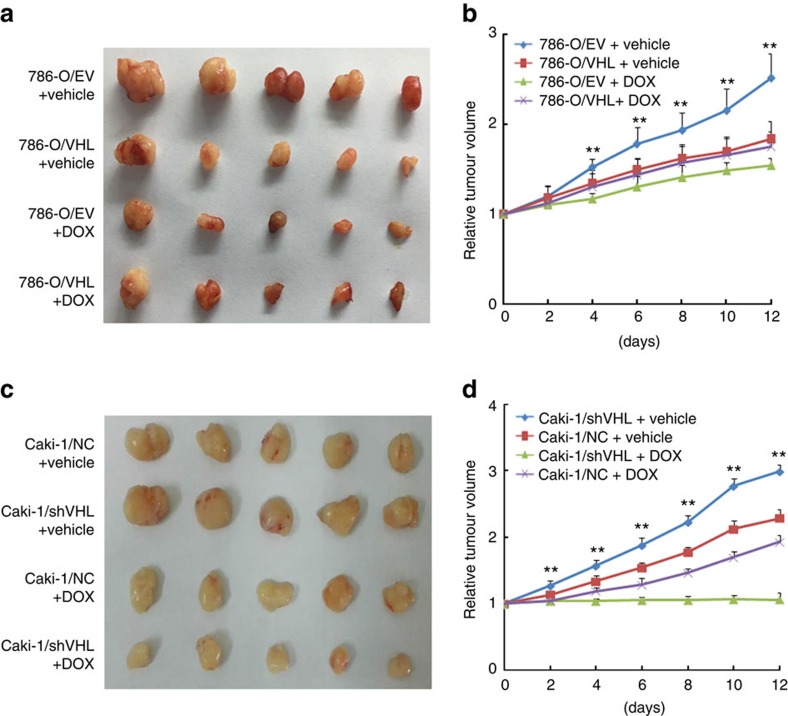
VHL-deficiency augments the sensitivity of ccRCC cells to anthracyclines *in vivo*. All indicated cells (5 × 10^6^) were injected into NOD/SCID mice. Tumour-bearing mice were treated every two days with vehicle or with 4 mg per kg doxorubicin by intraperitoneal injection. Five tumours per condition were analysed. (**a**,**c**) Pictures of tumour in NOD/SCID mice injected with the indicated cells. (**b**,**d**) Relative tumour volume growth. Data are mean±s.d. (*n*=5 for each group). (*P<0.05, **P<0.01 for *t*-test).

To evaluate the role of VHL on cytotoxicity of doxorubicin *in vivo*, 786-O/EV and 786-O/VHL or Caki-1/NC and Caki-1/shVHL cells were subcutaneously implanted into the flanks of NOD/SCID mice. As depicted in Fig. 2, doxorubicin treatment significantly reduced tumour growth of VHL-deficient tumour cells compared with corresponding VHL-expressing tumour cells (Fig. 2a–d). The same results were demonstrated in immunodeficient nude mice (Supplementary Fig. 2a–d). Collectively, all these data propose that VHL-deficient ccRCC cells present higher sensitivity to anthracyclines than that of VHL-proficient cells.

### HIFs do not contribute to the anthracycline cytotoxicity

**Figure 3:**
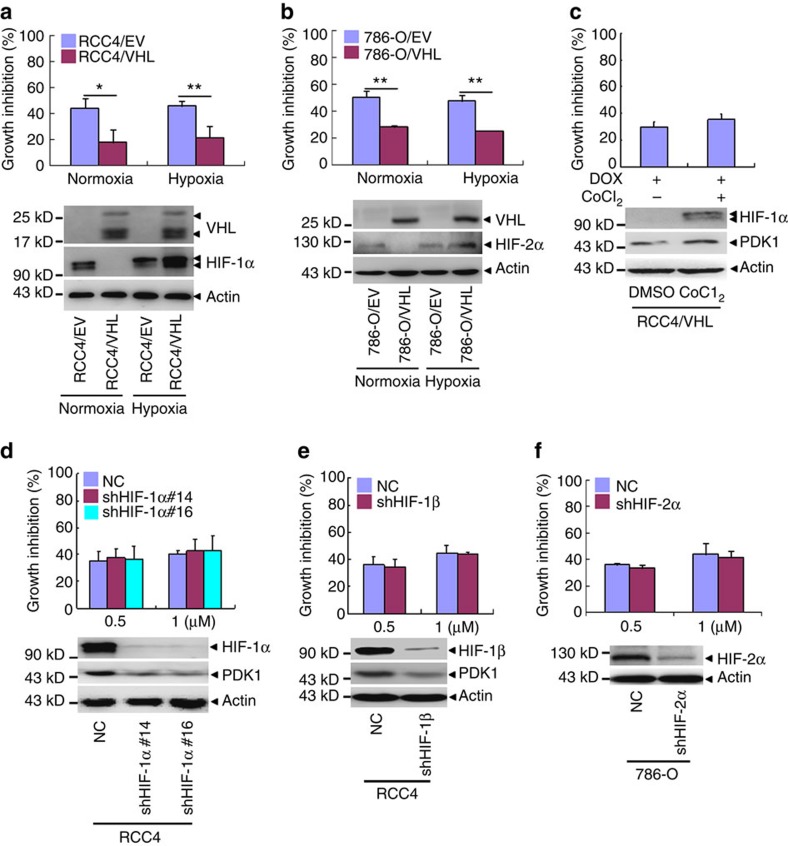
HIFs is not involved in cytotoxicity of anthracyclines in ccRCC cells. (**a**–**f**) The cell growth inhibition rates of indicated cells were detected by CCK-8 (top). The indicated proteins were detected by western blot (bottom). RCC4/EV and RCC4/VHL cells (**a**), or 786-O/EV and 786-O/VHL cells (**b**) were incubated in normoxia or hypoxia for 24 h followed by 1 μM doxorubicin treatment for 24 h. (**c**) RCC4/VHL cells were treated by 200 μM CoCl_2_ for 12 h and 0.1% DMSO as control, then treated with 1 μM doxorubicin for 48 h. (**d**–**f**) The indicated cells were treated with 0.5 or 1 μM doxorubicin for 24 h. (**d**) RCC4 cells were stably transfected with shRNAs against HIF-1α (α14 and α16). (**e**) HIF-1β was silenced by shRNAs in RCC4 cells. (**f**) 786-O cells were stably transfected with shRNAs against HIF-2α. Columns, means of three independent experiments with triplicate samples; bars, s.d. (*P<0.05, **P<0.01 for *t*-test).

Since HIF-α is a major substrate of VHL^4^,^32^, the potential role of HIFs in the regulation of cytotoxicity of anthracycline was tested. Towards this end, doxorubicin was applied to RCC4 and 786-O cells in the presence or absence of ectopic VHL expression under normoxia or hypoxia. Compared with normoxia, hypoxia, which accumulated HIF-1α and -2α proteins, failed to impact the drug cytotoxicity (Fig. 3a,b, Supplementary Fig. 3a). In addition, the cell growth inhibition after doxorubicin treatment had no significant difference in the presence or absence of hypoxia mimetic agent CoCl_2_ (Fig. 3c), which also accumulated the HIF-1α protein^33^. These results suggest that HIFs might not contribute to the enhanced cytotoxicity of anthracyclines. To consolidate this, we effectively silenced HIF-1α and HIF-1β or HIF-2α by their specific shRNAs in RCC4 or 786-O cells. Although these silences suppressed the proliferation (Supplementary Fig. 3b,c), they did not impact on the growth inhibition rates of doxorubicin in RCC4 or 786-O cells (Fig. 3d–f).

### Proteomics reveals regulators of anthracycline cytotoxicity

**Figure 4:**
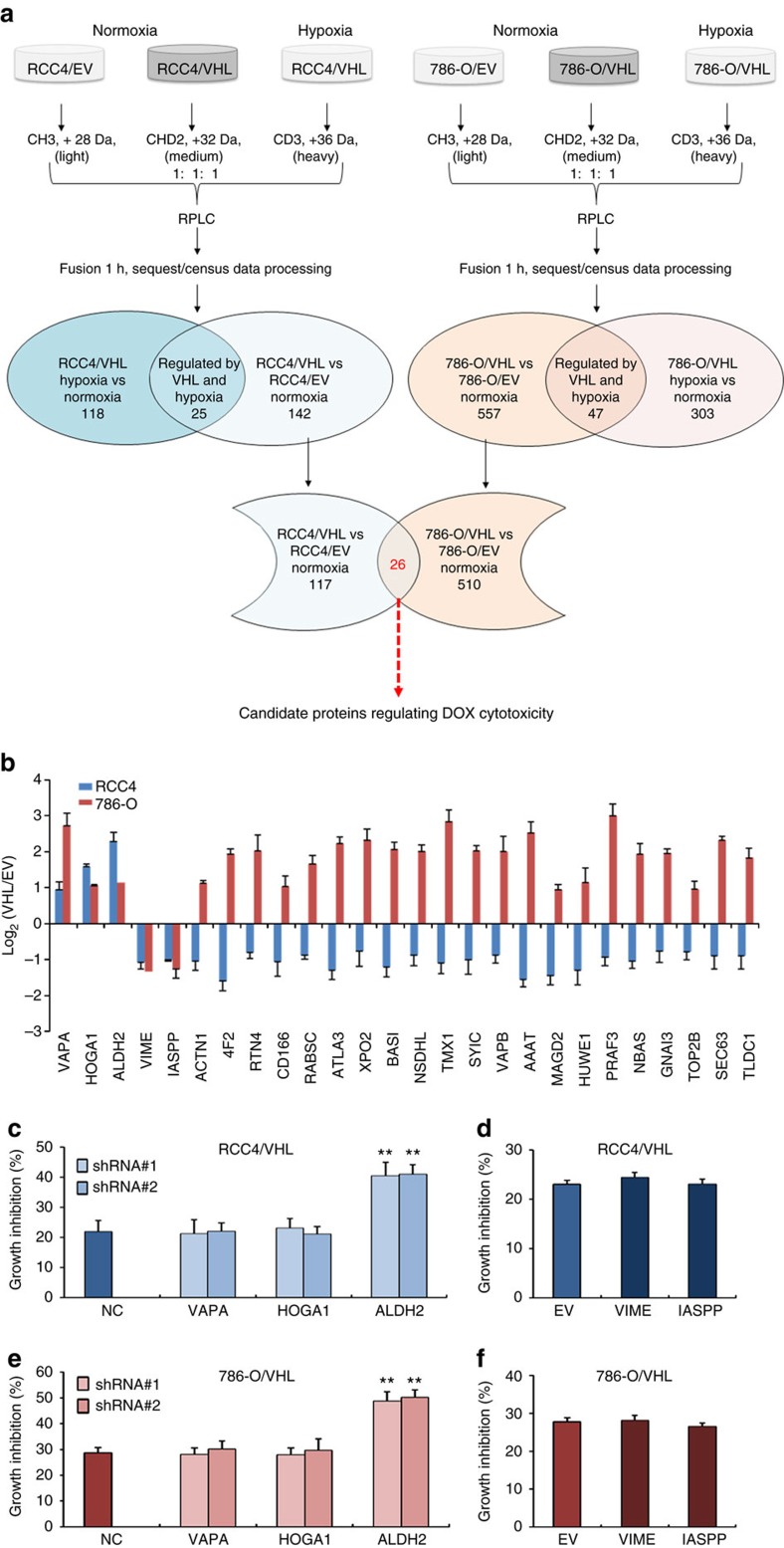
Subtractive proteomic analysis reveals candidate proteins regulating cytotoxicity of anthracyclines. (**a**) The schematic of proteomic work-flow. (**b**) The VHL alone-regulated proteins presented in both RCC4 and 786-O cells. The indicated genes were knocked down (**c**,**e**) or ectopically expressed (**d**,**f**) in RCC4/VHL (**c**,**d**) or 786/VHL (**e**,**f**) cells following by treatment with 1 μM doxorubicin for 48 h and the growth inhibition rates were detected. The indicated cells were compared to their counterpart (NC, non-specific shRNA control; EV, empty vector control) separately. Data represent means and s.d. of three independent experiments. (**P<0.01 for *t*-test).

Next, we sought to identify proteins mediating the enhanced cytotoxicity of anthracyclines in VHL-deficient RCC4 and 786-O cells by subtractive proteomics strategy. Figure 4a showed a schematic representation of the stable isotope dimethyl-labelling experiments. Briefly, cell lysates from each condition were digested and the peptides were labelled with light, medium and heavy reagents, respectively, and then were equally mixed and injected to Orbitrap Fusion liquid chromatography (LC)–mass spectrometry (MS/MS). Three independent experiments were performed in the indicated triplex configuration, which provided the direct comparisons between cells with EV and VHL transfection in normoxia, as well as cells with VHL transfection in normoxia and hypoxia.

As a result, we identified 4,282 and 3,585 proteins, respectively, in the pooled cell lysates of RCC4 and 786-O cells (Supplementary Data 1,2). All quantification data were normalized and natural log transformed before further analysis. Differentially expressed proteins were determined by applying paired *t*-test and corrected by Benjamini–Hochberg algorithm as multiple hypothesis testing correction^34^. Thus, 142 and 557 proteins were identified to be regulated, respectively, between RCC4/EV versus RCC4/VHL and 786-O/EV versus 786-O/VHL cells under normoxia (Supplementary Data 3,4). And 118 or 303 proteins were identified to be regulated in RCC4/VHL or 786-O/VHL cells under normoxia and hypoxia (Supplementary Data 5,6). Accordingly, 25 and 47 proteins were regulated by both VHL and hypoxia in RCC4 and 786-O cells (Supplementary Data 7,8). And thus, 117 and 510 proteins were identified to be regulated by VHL but not hypoxia, respectively, in RCC4 and 786-O cells (Supplementary Data 9,10). To get an overview of these VHL alone-regulated proteins in RCC4, function enrichment and pathways analysis were performed using QIAGEN’s Ingenuity Pathway Analysis tools. The bioinformatic analysis indicated complicated biological processes, such as organismal injury and abnormality, cancer, cell growth and proliferation, and multiple pathways were involved (Supplementary Fig. 4a–d).

The comparison of the bioinformatic data revealed that RCC4 and 786-O presented very different functional state (Supplementary Fig. 4a–d and Supplementary Data 11,12). The functional enrichment and pathway analysis suggested that RCC4 cells presented a series of suppressed functional state such as cellular movement, maintenance, cellular assembly and organization, whereas 786-O demonstrated a series of active functional state in the cellular processes mentioned above, which might contribute to the different biological characteristics of these two cell lines. 786-O was derived from a primary clear-cell adenocarcinoma and high tumorigenic, but RCC4 was low tumorigenic^35^,^36^.

However, these two VHL-deficient cell lines shared common effect on the enhanced cytotoxicity of doxorubicin. To further narrow down the candidate proteins involved in cytotoxicity of anthracyclines, these results of IPA analysis were compared between these two cell lines (Supplementary Data 11,12) and the common function enrichment and pathways were picked up (Supplementary Data 13,14). Totally, 107 function enrichment or pathway including cancer, cell death and survival, organismal injury and abnormalities were shared by RCC4 and 786-O cells (Supplementary Data 13,14). Among the proteins involved in these common function enrichment and pathway, 26 were shared by RCC4 and 786-O (Fig. 4a,b). Notably, five of these 26 VHL alone-regulated proteins, including vesicle-associated membrane protein-associated protein A, 4-hydroxy-2-oxoglutarate aldolase 1, aldehyde dehydrogenase 2 (ALDH2), vimentin and protein phosphatase 1 regulatory subunit 13 like, were found to be regulated consistently in RCC4 and 786-O cells (Fig. 4b). Hence, we silenced or ectopically expressed these five genes followed by doxorubicin treatment in RCC4/VHL (Fig. 4c,d) and 786-O/VHL (Fig. 4e,f). The results demonstrated that *ALDH2* but not other four genes regulated the cytotoxicity of doxorubicin in both ccRCC cells.

### ALDH2 regulates anthracycline cytotoxicity

**Figure 5:**
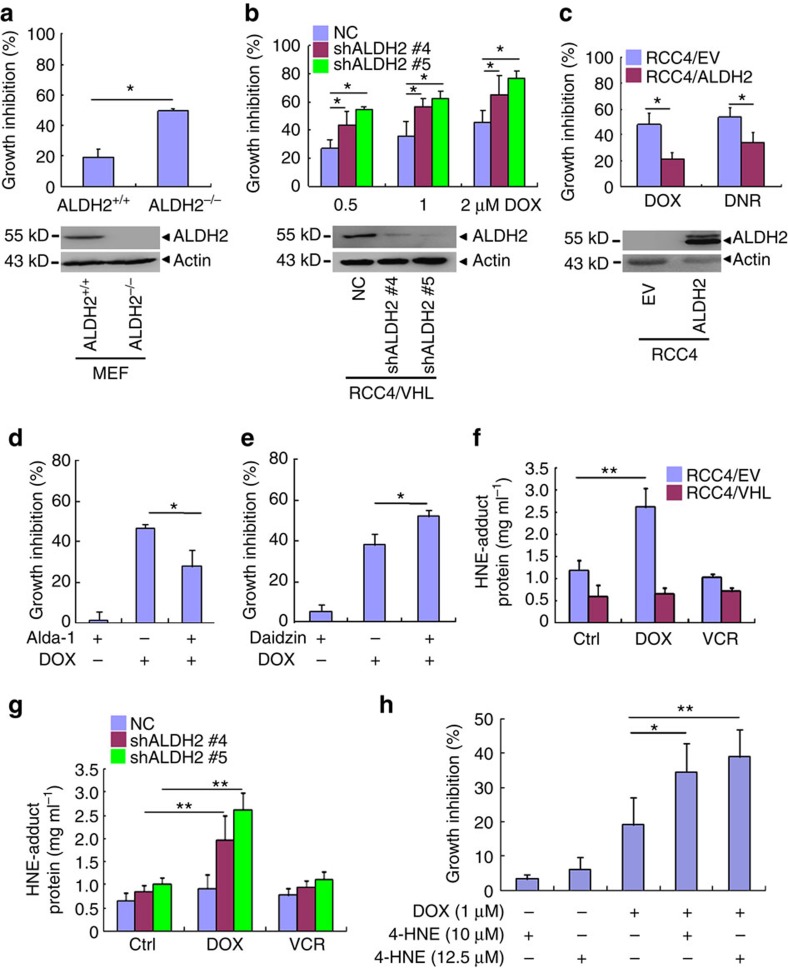
ALDH2 mediates the enhanced cytotoxicity of anthracyclines in VHL-deficient ccRCC cells. (**a**–**c**) The cell growth inhibition rates of the indicated cells (top) and the indicated proteins were detected by western blot (bottom). (**a**) The primary embryo fibroblast (MEF) cells from ALDH2 knockout mice. ALDH2^+/+^ and ALDH2^−/−^ represent MEF cells from ALDH2 wild type and knockout mice, then treated by 1 μM doxorubicin for 24 h. (**b**) RCC4/VHL cells were stably transfected with shRNAs against ALDH2 (#4 and #5), then treated with 1 μM doxorubicin for 48 h. (**c**) RCC4 cells were stably transfected with ALDH2 expression vector and treated by 1 μM doxorubicin or daunomycin for 24 h. (**d**,**e**) The growth inhibition rates of RCC4/VHL cells treated by indicated drugs. (**d**) RCC4/VHL cells were pretreated with 50 μM ALDH2 activator alda-1 for 1 h, then treated in combination with 1 μM DOX for 48 h. (**e**) Pretreatment with 60 μM ALDH2 inhibitor daidzin for 24 h, then treated RCC4/VHL cells in combination with 1 μM DOX for 48 h. (**f**,**g**) HNE-adduct protein levels of the indicated cells treated by 1 μM DOX or VCR for 24 h. (**h**) The growth inhibition rates of RCC4/VHL cells treated by DOX and/or 4-HNE for 24 h. Columns, means of three independent experiments; bars, s.d. (*P<0.05,**P<0.01 for *t*-test).

ALDH2 was reported to regulate cytotoxicity of doxorubicin in cardiac cells, leukaemia cells and lung cancer cells^37^,^38^,^39^. Indeed, the cytotoxicity of doxorubicin was significantly higher in the primary mouse embryo fibroblast (MEF) cells from ALDH2 knockout mice than those from wild-type mice (Fig. 5a). To address the potential role of ALDH2 in cytotoxicity of anthracyclines in ccRCC cells, we silenced ALDH2 expression in RCC4/VHL cells, and found that the reduction of ALDH2 suppressed the proliferation of these cells (Supplementary Fig. 5a), although the ALDH2 knockout did not affect the proliferation of the primary MEF cells (Supplementary Fig. 5b). Then, these RCC4/VHL cells were treated with doxorubicin at different concentrations, and showed that cytotoxicity of doxorubicin significantly increased in ALDH2 silencing cells (Fig. 5b). Reciprocally, stable ectopic expression of ALDH2 decreased cytotoxicity of doxorubicin and daunorubicin in RCC4 cells (Fig. 5c). Because cytotoxicity of doxorubicin is contributed partially by apoptosis^30^, the apoptosis of these cells was examined. The doxorubicin-treated RCC4/EV cells had a higher apoptosis rate than RCC4/VHL (Supplementary Fig. 5c), and cell apoptosis rate was higher in the ALDH2^−/−^ MEF cells (Supplementary Fig. 5d) and RCC4/VHL with ALDH2 silencing (Supplementary Fig. 5e).

To explore whether ALDH2 enzyme activity affected doxorubicin-treated cells, we used ALDH2 activator alda-1 (ref. 23) and inhibitor daidzin^40^ to pre-treat RCC4/VHL cells. The results showed that alda-1 reduced, while daidzin increased, the cytotoxicity of doxorubicin in RCC4/VHL cells (Fig. 5d,e), suggesting that doxorubicin cytotoxicity might be negatively correlated with enzymatic activity of ALDH2.

It has been reported that doxorubicin could increase the level of intracellular ROS and 4-HNE (ref. 29). As the main metabolism enzyme of 4-HNE, ALDH2 may affect cell deaths by metabolism of 4-HNE. In agreement, ectopic VHL expression significantly reduced 4-HNE in RCC4 cells, while ALDH2 silence increased 4-HNE in the RCC4/VHL cells, under the treatment of doxorubicin but not vincristine (Fig. 5f,g). Furthermore, doxorubicin combined with 4-HNE led to even higher growth inhibition than doxorubicin treatment alone in RCC4/VHL cells (Fig. 5h). All these data propose that a higher intracellular 4-HNE levels endow cells with higher cytotoxicity of doxorubicin.

### VHL regulates the transcription of ALDH2

**Figure 6:**
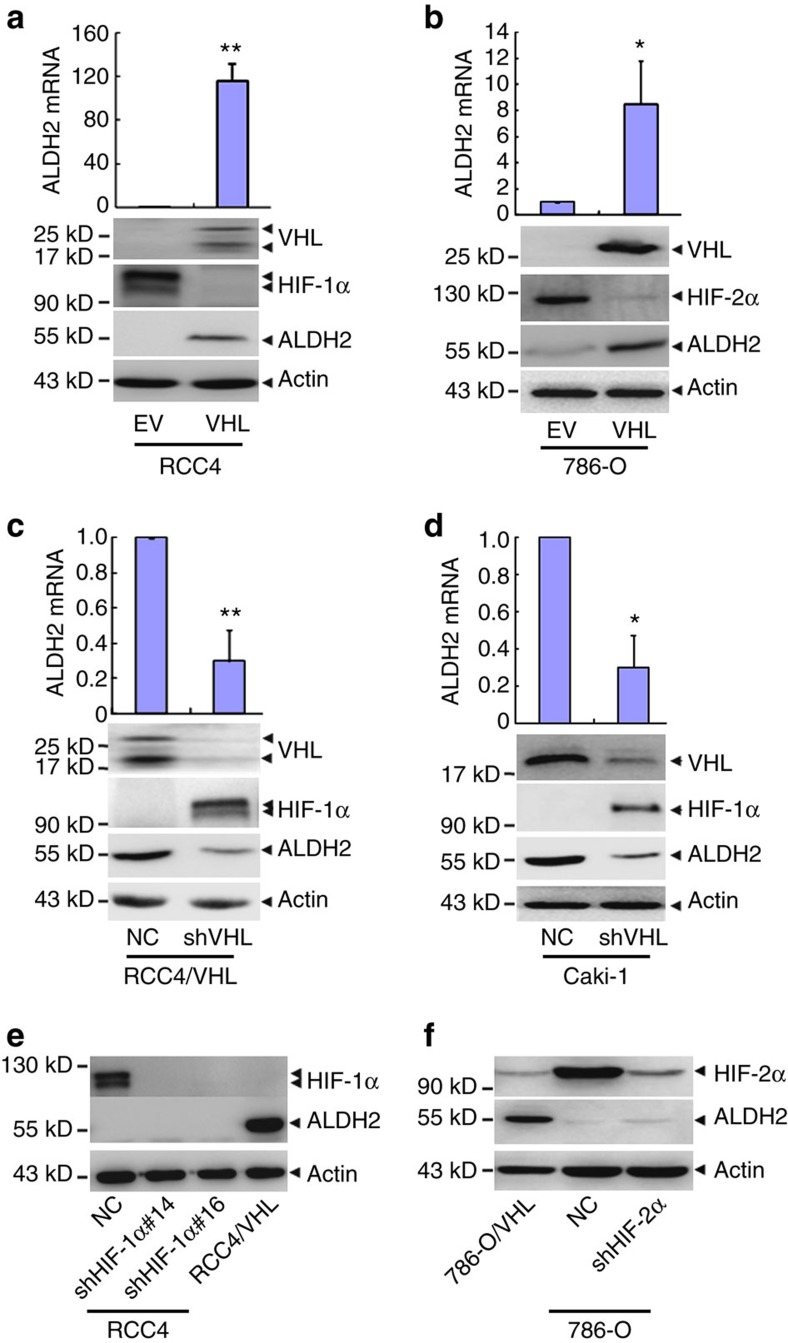
VHL regulates ALDH2 in a HIF-independent manner. (**a**–**f**) The mRNA and/or protein levels of the indicated genes were detected by real-time quantitative RT–PCR and western blot respectively. Actin was used as the loading control. RCC4 cells (**a**) and 786-O (**b**) were stably transfected with VHL expression vector (VHL) or empty vector (EV). RCC4/VHL (**c**) and Caki-1 cells (**d**) were infected with retroviral vectors harbouring shRNA against VHL (shVHL) or non-specific control (NC). (**e**) RCC4 cells were stably transfected with shRNAs against HIF-1α (α14 and α16). (**f**) 786-O cells were infected with retroviral vectors harbouring shRNA against HIF-2α (shHIF-2α) or non-specific control (NC). Columns, means of three independent experiments; bars, s.d. (*P<0.05,**P<0.01 for *t*-test).

To validate the proteomic results that VHL regulates ALDH2 in ccRCC, *VHL* was overexpressed or knocked down in ccRCC cells. As shown in Fig. 6a, the VHL overexpression in RCC4 augmented the expression of ALDH2 in mRNA and protein levels. This is also true in 786-O cells (Fig. 6b). Visa versa, both mRNA and protein of ALDH2 were significantly downregulated by silencing VHL expression in RCC4/VHL and Caki-1 (Fig. 6c,d). Consistent with the above findings that HIFs do not contribute to the enhanced cytotoxicity of anthracyclines in VHL-deficient ccRCC cells, the ALDH2 protein levels were also not affected by HIF-1α and HIF-2α silencing in RCC4 cells and 786-O cells (Fig. 6e,f). These results suggest that VHL regulates the transcription of ALDH2 in ccRCC cells in HIF-independent manner.

As documented^4^,^41^, the ubiquitin E3 ligase activity of VHL is dependent upon its α-domain and β-domain, among which the β-domain directly binds putative substrates such as HIF-1α, while the α-domain directly contacts elongin C in pVHL-elongin C- elongin B complex. We constructed an α-domain mutant C162F and a β-domain mutant Y98H of VHL, which lost its E3 ligase activity^41^, to examine the expression of ALDH2. The results showed that, like wild-type VHL, these two VHL mutants still upregulated ALDH2 expression, but these mutants failed to induce HIF-1α degradation (Supplementary Fig. 6a). Of great interest, we sequenced *VHL* genes from 44 cases of ccRCC cancer tissues, and found that 13 cases of them carried VHL mutations, among which 8 cases were missense mutations with no E3 ligase activities (Supplementary Fig. 6b). In spite of this, tissues with wild type and mutated VHL had the similar levels of ALDH2 protein, as evaluated by immunohistochemistry (Supplementary Fig. 6c–e). Cumulatively, our results suggest that VHL regulates the transcription of ALDH2 in an E3 ubiquitin ligase-independent manner.

### HNF-4α mediates regulation of ALDH2 by VHL

**Figure 7:**
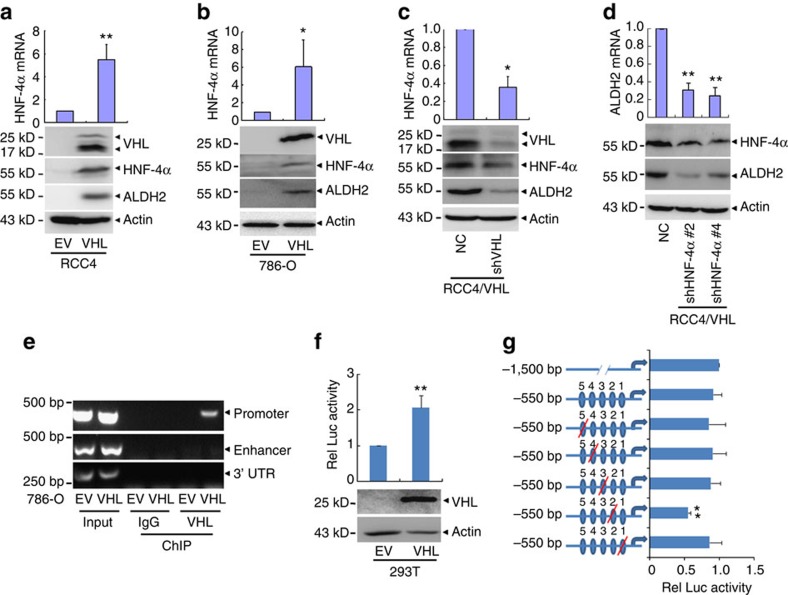
VHL regulates ALDH2 expression through HNF-4α. (**a**–**c**) The mRNA and protein levels of the indicated genes were detected by real-time quantitative PCR with reverse transcription (RT–PCR) and Western blot respectively. (**d**) RCC4/VHL cells were stably transfected with shRNAs against HNF-4α (#2 and #4), the mRNA and protein levels of the indicated genes were detected. (**e**) VHL binding to HNF-4α promoter or enhancer was analysed by chromatin immunoprecipitation in 786-O/EV and 786-O/VHL cells. Chromatin was immunoprecipitated with IgG or anti-VHL antibody and analysed by PCR using primers indicated above. (**f**) 293T cells were transfected with luciferase reporter plasmids driven by HNF-4α promoter together with or without VHL expression vector for 36 h, then detected the relative luciferase activity. Protein levels of VHL were detected by western blot with actin as a loading control (low panel). (**g**) Luciferase reporter plasmids driven by trunk or mutated sequences as indicated were transfected together with VHL-expressing vector or empty vector into 293T cells for 36 h. All the relative luciferase activities of HNF-4α promoter were normalized by pSV40-Renilla and estimated as the relative folds against cells. Blue ovals represent potential-binding sites. Arrows represent the transcriptional start point of HNF-4α. Columns, means of three independent experiments; bars, s.d. (*P<0.05,**P<0.01 for *t*-test).

It was reported that hepatocyte nuclear factor 4α (HNF-4α) is a transcription factor of ALDH2 (refs 42, 43). Although HNF-4α was not detected in the proteomic analysis for its low abundance as a transcription factor, we put HNF-4α as well as all the proteins regulated by VHL but not hypoxia into the protein interaction analysis with IPA. Indeed, HNF-4α was found to be a central node in the network in RCC4 (Supplementary Fig. 7a) and 786-O (Supplementary Fig. 7b). In addition, several HNF-4α target genes were found in these VHL alone-regulated genes (Supplementary Data 15). Thus, we extrapolated that HNF-4α might be involved in the regulation of ALDH2 by VHL. To confirm this, we showed that HNF-4α mRNA and protein levels were significantly upregulated in RCC4 and 786-O cells with ectopic VHL expression (Fig. 7a,b). Accordingly, silencing of VHL in RCC4/VHL cells suppressed HNF-4α expression on mRNA and protein levels (Fig. 7c). On the other hand, silencing of HNF-4α decreased ALDH2 expression in RCC4/VHL (Fig. 7d). Our results suggest that VHL could upregulate the mRNA and protein levels of HNF-4α to activate ALDH2 transcription.

Furthermore, we found that anti-VHL antibody but not normal mouse IgG could precipitate the putative promoter of *HNF-4α* (Fig. 7e). Hence, *VHL*-expressing plasmids and the 1,500 bp of the *HNF-4α* promoter-driven luciferase reporter were co-transfected into 293T cells, and the results demonstrated that *VHL* transfection significantly increased the luciferase activity (Fig. 7f). In addition, we constructed 550 bp trunk of the *HNF-4α* promoter, and five different mutants of this trunk^39^,^44^. The second mutant, from −130 bp to −160 bp, significantly decreased the luciferase activity in response to *VHL* transfection (Fig. 7g), which indicate that this site is essential for VHL regulating the transcription of HNF-4α.

### VHL is positively correlated with HNF-4α and ALDH2

**Figure 8:**
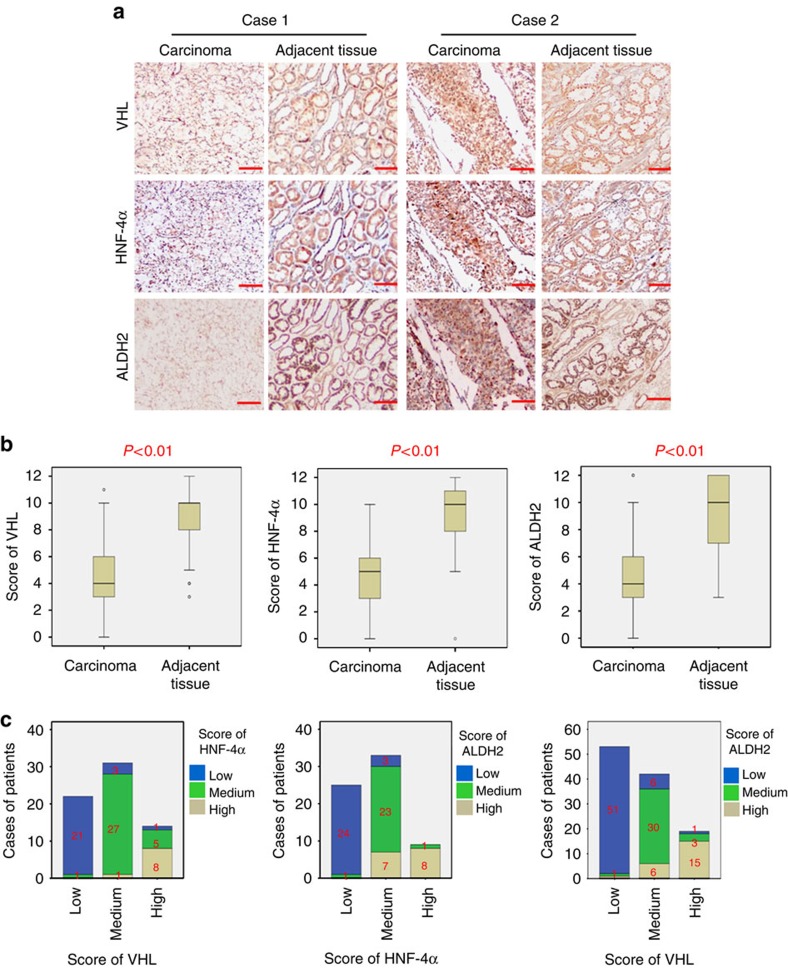
Correlation of these proteins in ccRCC tissues. (**a**) Representative immunohistochemistry images of ccRCC samples for the low and high expressions of indicated proteins. The scale bar represents 100 μm. (**b**) VHL, HNF-4α and ALDH2 expression scores are shown as box plots, with the horizontal lines representing the median; the bottom and top of the boxes representing the 25th and 75th percentiles, respectively; and the vertical bars representing the range of data. We compared renal clear cell carcinoma tissues with matched adjacent tissues (*n*=114). Any outliers are marked with a circle. *P* value is calculated via *t*-test. (**c**) Correlation analysis of VHL, HNF-4α and ALDH2 expression are calculated via Pearson’s *χ*^2^ test and all the *P* value are less than 0.01.

We further explored whether the relationships among VHL, HNF-4α and ALDH2 existed in clinical patient samples. As for this, 114 cases of RCC samples were collected to detect the expression of VHL, HNF-4α and ALDH2 in cancer and adjacent tissues by immunohistochemical assay. Significantly lower expression of VHL, HNF-4α and ALDH2 were found in cancer tissues than their corresponding adjacent tissues (Fig. 8a,b). On the other hand, as shown in Fig. 8c, high-VHL expression was often associated with high HNF-4α and ALDH2 expression, and vice versa (*P*<0.01). Taken together, these results suggest that VHL protein expression has a positive correlation with HNF-4α and ALDH2 in ccRCC.

Furthermore, 72 cases of ccRCC patients with cancer and corresponding adjacent tissues in database GEO were used to verify the relationship among VHL, HNF-4α and ALDH2. As shown in Supplementary Fig. 8a–c, lower mRNA expression of VHL, HNF-4α and ALDH2 were found in cancer tissues compared to their corresponding adjacent tissues, and there was a positive correlation between VHL and HNF-4α mRNA (Supplementary Fig. 8d) and between HNF-4α and ALDH2 mRNA (Supplementary Fig. 8e). But we could not find a correlation between VHL and ALDH2 mRNA (Supplementary Fig. 8f), which might be because VHL functional mutations interfere with protein stability or the alteration of the VHL start codon could result in loss of VHL protein expression^45^,^46^.

### HNF-4α mediates the cytotoxicity of anthracyclines

**Figure 9:**
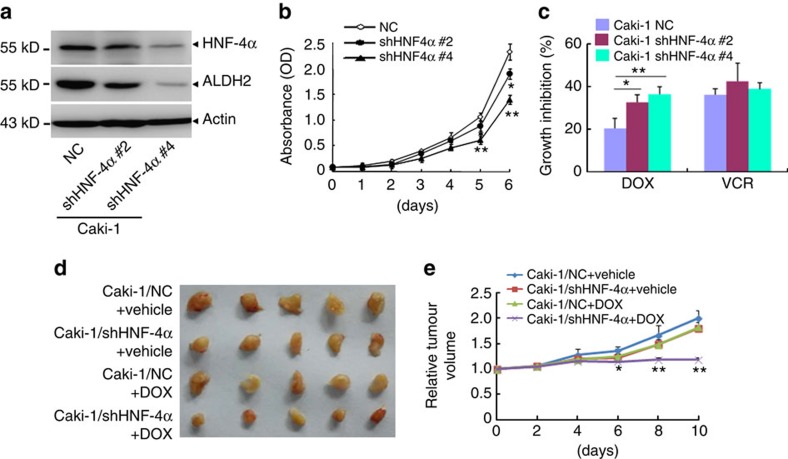
HNF-4α regulates cytotoxicity of anthracyclines in ccRCC. (**a**) Caki-1 cells were stably transfected retroviral vectors harbouring shRNA against HNF-4α (#2 and #4) or non-specific control (NC). Then the indicated proteins were detected by western blot. (**b**) Growth curves of the indicated cells. (**c**) The indicated cells were treated with 1 μM doxorubicin for 24 h and the growth inhibition rates were detected. (**d**,**e**) All indicated cells (5 × 10^6^) were injected into nude mice. Tumour-bearing mice were treated every 2 days with vehicle or with 4 mg per kg doxorubicin by intraperitoneal injection. Five tumours per condition were analysed. (**d**) Pictures of tumours in nude mice injected with the indicated cells. (**e**) Relative tumour volume growth. Data are mean±s.d. (*n*=5 for each group). (*P<0.05, **P<0.01 for *t*-test).

To further confirm the role of HNF-4α in the cytotoxicity of anthracyclines in ccRCC, we silenced the expression of HNF-4α in Caki-1 cells by shRNA specifically against HNF-4α (Fig. 9a). The results demonstrated that the silence inhibited the cell proliferation (Fig. 9b), and significantly increased the cytotoxicity of doxorubicin (Fig. 9c). We also subcutaneously implanted Caki-1/NC and Caki-1/shHNF-4α cells into the flanks of nude mice. Tumour growth of Caki-1/shHNF-4α cells were significantly reduced compared with that of Caki-1/NC cells after doxorubicin treatment (Fig. 9d,e). Collectively, HNF-4α mediates the cytotoxicity of anthracyclines in ccRCC.

### Regulation of anthracycline toxicity by VHL is conserved

**Figure 10:**
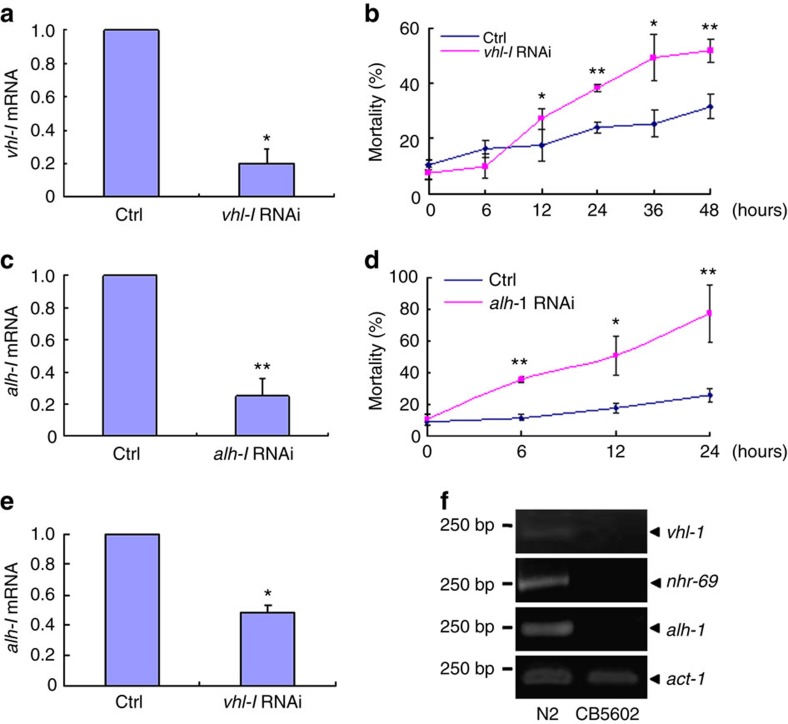
Regulation of anthracycline toxicity and ALDH2 expression by VHL are conserved. (**a**)The mRNA of *vhl-1* in *vhl-1* RNAi *C.elegans*. (**b**) The mortality of indicated *vhl-1* RNAi *C.elegans* treated by 20 μg ml^−1^ doxorubicin for different time. (**c**) The mRNA of *alh-1* in *alh-1* RNAi *C.elegans*. (**d**) The mortality of indicated *alh-1* RNAi *C.elegans* treated by 20 μg ml^−1^ doxorubicin for different time. (**e**) The mRNA of *alh-1* detected by semi-quantitative RT–PCR in *vhl-1* RNAi *C.elegans*. (**f**) The mRNA expression of indicated genes detected by semi-quantitative RT–PCR in *vhl-1* deficiency *C.elegans* CB5602. Ctrl represents control. RNAi represents RNA interference. Twenty-five cycles used for semi-quantitative RT–PCR. Columns, means of three independent experiments; bars, s.d. (*P<0.05, **P<0.01 for *t*-test).

VHL is a conservative key regulator under hypoxia condition. We further investigated its conservation on regulation of ALDH2 and chemotherapeutic toxicity. *C.elegans* is good system to verify the conservation of gene function as well as drug action^47^. The double-mutant strain of *C.elegans acs-20;acs-22* increases penetration of several drugs^47^ but has no effect on worm survivability. Thus this mutant worms were used to test the anthracycline toxicity and the role of these genes. As shown in Supplementary Fig. 9a, this mutant had no effect on the survival of the worms when no drugs were applied. Of note, this mutant strain of *C.elegans* had a higher toxicity of doxorubicin than its wild-type strain (Supplementary Fig. 9b). In addition, the RNAi of *vhl-1* (corresponding to human VHL) and *alh-1* (corresponding to human ALDH2) had no effect on the survival to this mutant worms (Supplementary Fig. 9c,d). Consistent to our findings in ccRCC cell lines, the silence of either *vhl-1* or *alh-1* significantly increased the toxicity of doxorubicin in *C.elegans* compared with treatment of DMSO with the same osmolality (Fig. 10a–d). Furthermore, *vhl-1* silencing also inhibited *alh-1* mRNA expression (Fig. 10e). Intriguingly, loss of function *vhl-1* mutant could downregulate the mRNA of *nhr-69* (HNF-4α in mammal) and *alh-1* in *C.elegans* (Fig. 10f). Collectively, these results indicate the evolutionary conservation in the regulation of ALDH2 and HNF-4α expression and the anthracyclines toxicity by VHL.

## Discussion

Here we found that VHL-deficiency made RCC cells more sensitive to anthracyclines. Previously, VHL overexpression has been found to synergize with doxorubicin to suppress hepatocellular carcinoma in mice^48^, suggesting different effects of VHL on anthracycline response in different cellular context between liver and kidney. Considering that HIF-α is the major substrate of VHL, we investigated whether HIFs are involved in the effect of VHL on anthracycline sensitivity in ccRCC cells. It has been reported that suppression of HIF-2α restores P53 activity and promotes the apoptosis induced by anthracyclines^49^, and it has also been reported that anthracyclines inhibited the binding of HIF heterodimer to the consensus HRE and thus impaires the transcriptional response of HIF^50^. However, our results show that the VHL-deficiency increased sensitivity of ccRCC cells to anthracyclines is independent upon HIF-1 or HIF-2.

We demonstrate that *ALDH2* is a target gene of VHL and participates in cytotoxicity of anthracyclines in ccRCC. The regulation of cytotoxicity of anthracyclines in cardiac cells by ALDH2 has been reported by several studies recently. DOX-induced myocardial cellular toxicity has been found to be further aggravated in ALDH2 knockout mice and ameliorated in ALDH2 transgenic mice^37^. Consistently, the cardiotoxicity is also found to be aggravated when the DOX plus ALDH2 inhibitor daidzin, while DOX plus ALDH2 agonist Alda-1 would partial or complete alleviate the cardiotoxity^39^. Notably, Moreb *et al*. have found that overexpression of ALDH2 in leukaemia cell K562 and lung cancer-cell H1299 could promote their proliferation and resistant to doxorubicin^38^. These findings suggested the sensitivity to anthracyclines is regulated by ALDH2, which is consistent to our finding in RCC cells. Considering the fact that an inactive mutant form of ALDH2 is found in 40% of East Asian populations^19^, it is of great interest to explore whether these findings can be extended to other cancers in the future.

Our further investigation show that the effect of VHL on cytotoxic sensitivity to anthracyclines in ccRCC cells is not dependent on its E3 ligase activity. VHL is located in both cytoplasm and nucleus, the nuclear form of VHL exhibits the anti-tumour properties^51^. There are a few reports that VHL plays a role in other ways except as an E3 ubiquitin ligase. It has been reported that VHL mediates the transcriptional suppression of the *c-Myc* gene by binding to the *c-Myc* promoter^52^. More intriguingly, here we also show that VHL could activate HNF-4α transcription through binding to the promoter of *HNF-4α* and revealed a novel nuclear function of VHL.

As a member of HNF-4, HNF-4α belongs to subfamily NR2 of the nuclear receptor superfamily^53^. HNF-4α is a key transcription factor for hepatocyte differentiation, proliferation control, cellular homeostasis, epithelial morphogenesis, glucose metabolism and insulin secretion^54^,^55^,^56^,^57^,^58^. Reduced expression of HNF-4α is found in hepatocarcinogenesis and confers advantages to tumour cells^59^,^60^,^61^. Thus, HNF-4α could be considered as a potential tumour suppressor in liver cells. The malfunction of HNF-4α has also been described in human RCCs^62^. Our results indicate that VHL could regulate HNF-4α, which partially explains the low expression of HNF-4α in RCC.

Despite the limitations in detecting low-abundance proteins, proteomics could reveal the most significant changes of high-abundance proteins through an unbiased strategy, which could demonstrate some of the most important aspects of the drug effects. In this sense, proteomics could accelerate the drug development via uncovering the action mechanism of novel reagents^63^. On the other hand, proteomics, as drug targets discovery means, always encounter a bottleneck to conceive the real targets. Functional analysis of potential drug target proteins will be great helpful.

As a model for drug target verification, *Caenorhabditis elegans* has its advantage to verify the functional conservation^64^. However, the surface barrier is essential for maintaining the internal environment to *C.elegans* and prevents drugs penetrating into the bodies of *C.elegans*^65^. *acs-20;acs-22* mutant *C.elegans* has defective skin barriers, and thus drugs can easily penetrate into the bodies of *C.elegans*, which greatly reduces the lethal dose of drugs^47^. In our work, we have verified the drug action and candidate genes affecting cytotoxicity of anthracyclines by *acs-20;acs-22* mutant and demonstrated the value of this mutant worm.

Conclusively, we found that VHL-deficiency augments anthracycline chemotherapy by downregulation of ALDH2 in ccRCCs. This work might provide clues for understanding the novel function of VHL and precise treatment for ccRCC patients.

## Methods

### Cell culture and treatment

A498, ACHN, 786-O, HK-2, 769-P, OS-RC-2, Caki-1, Caki-2 were purchased from cell bank of Chinese Academy of Science, Shanghai. RCC4, RCC4/EV and RCC4/VHL were provided by Dr J.K. Cheng in SJTU-SM. There were no signs of mycoplasma contamination for all cell lines. RCC4, RCC4/EV and RCC4/VHL were cultured in DMEM medium (Invitrogen) supplemented with 10% FBS (Gibco BRL, Gaithersburg, MD, USA). A498, ACHN were cultured in MEM medium (Invitrogen) with 10% FBS, 786-O, HK-2, 769-P, OS-RC-2 were cultured in RPMI-1640 medium with 10% FBS, Caki-1and Caki-2 were cultured in McCoy’5A (Invitrogen) with 10% FBS. The cell lines were cultured in 5% CO_2_/95% air in a humidified atmosphere at 37 °C. Hypoxic treatment was performed in a specially designed hypoxia incubator (Thermo Electron, Forma, MA, USA) with 1% O_2_, 5% CO_2_ and 93% N_2_.

### Tissue samples and immunohistochemistry

Paraffin-embedded tumour tissues and normal adjacent tissues from Ruijin Hospital. The immunohistochemical analysis was performed on the 4 μm thick fraction mounted on charged slides and sectioned from each clinical sample. Then, each slide was deparaffinized in 60 °C, followed by treatment with xylene and graded alcohol. After the antigen retrieval and being blocked with 5% bovine serum albumin, tissue slides were immunohistochemically stained by antibodies against VHL (1:50, Abcam, ab140989), HNF-4α (1:50, Abcam, ab181604) and ALDH2 (1:200, Abgent, AM1831a), respectively, then visualized by standard avidin-biotinylated peroxidase complex method. Then, hematoxylin was used for counterstaining and morphologic images were observed with Olympus BX51 microscope. All of staining was assessed by pathologists blinded to the origination of the samples and subject outcome. Each specimen was assigned a score according to the intensity of the staining (no staining=0; weak staining=1, moderate staining=2, strong staining=3) and the extent of stained cells (0%=0, 1–24%=1, 25–49%=2, 50–74%=3, 75–100%=4). The final immunoreactive score was determined by multiplying the intensity score with the extent of score of stained cells, ranging from 0 (the minimum score) to 12 (the maximum score).

### Quantitative real-time PCR

Total RNA was isolated by TRIzol reagent (Invitrogen, Carlsbad, CA, USA) and treated with RNase-free DNase (Promega, Madison, WI, USA). Reverse transcription was performed with TaKaRa RNA PCR kit (TaKaRa, Dalian, China). The double-stranded DNA dye SYBR Green PCR Master Mixture Reagents (Applied Biosystems, Warrington, UK) was used for quantitative real-time reverse transcription–polymerase chain reaction (PCR) analysis. The following specific primers used were 5′-TGGTGGACAAAGACAAGAGG-3′ (forward) and 5′- AGGAGCGCATTGATGGAG-3′ (reverse) for HNF-4α, 5′-TTCGCCCTGT TCTTCAACCA-3′ (forward) and 5′-CCTGCTCGGTCTTGC TATCAAA-3′ (reverse) for ALDH2, 5′-CTGGCCTCTGCCATCTTCTG-3′ (forward) and 5′-TTAGC CTCCTTGCTCACATGC-3′ (reverse) for CYP1A2, and 5′-CATCCTCACCCTGAAGTACCC-3′ (forward) and 5′-AGCCTGGATAGCAA CGTACATG-3′ (reverse) for Actin as control. To *C. elegans*, 5′-ACGGCATAA TCCAACTGA-3′ (forward) and 5′-AGGAGGAGGAATTGAACG3′ (reverse) for vhl-1, 5′- TCGGCAGTGAGTGGAGAC-3′ (forward) and 5′-CGGCGTAATA AVGAAGAGT-3′ (reverse) for alh-1, 5′-AGCGGGAATGAAGAGTA-3′ (forward) and 5′-CGTATGGTGCAAGTGAAG -3′ (reverse) for nhr-69, 5′-CCATCATGAAGTGCGACATTG-3′ (forward) and 5′-CATGGTTGATGGG GCAAGAG-3′(reverse) for act-1 as control. The folds of changes were shown as means±s.d. in three independent experiments with each triplicate.

### shRNA design and transfection

Pairs of complementary oligonucleotides against VHL were synthesized, annealed and ligated into pSIREN-RetroQ according to the manufacturer’s instruction (Clontech, Mountain View, CA, USA). The target sequence for VHL was 5′-GAGCCTAGTCAAGCCTGAG-3′, the sequences for ALDH2 were 5′-TTATATCACCATTAAGGCA-3′ and 5′-ATGTCTCCGGTATTATGCC-3′, the sequences for HNF-4α were 5′-AAGGTCAAGCTATGAGGACAG-3′ and 5′-AAGCAGGAAGTTATCTAGCAA-3′, and the sequences for HIF-1α were 5′-GGACAGTACAGGATGCTTGC-3′ and 5′-GGTGGATTACCACAGCTGAC-3′ (ref. 12). After transfection for 48 h, the viral supernatant was collected, filter-sterilized and added to cells in six-well plate containing polybrane with a final concentration of 4 μg ml^−1^ and then puromycin (2 μg ml^−1^) was added to select the stably transfected cells after another 48 h.

### Cytotoxicity assay and cell proliferation

For cytotoxicity assays, 5,000 ccRCC cells were plated in 96-well plates in 100 μl of media, respectively. The following day, different concentrations of drugs was added in media for different time points at 37 °C. Then each well was pulsed by addition of 10 μl of CCK-8 assay (WST-8; Cell Counting Kit-8 from Dojindo, Kumamoto, Japan) and incubated for 3 h. Absorbance readings at a wavelength of 450 nm were taken on Synergy H4 Hybrid Microplate Reader. The growth inhibition rate is calculated by (Ac–Ae)/(Ac–Ab) × 100%. Ac, Ae and Ab mean absorbance value in control, experiment (Ae) and blank.

Cell proliferation was also evaluated by the CCK-8 assay. Briefly, 1,000 cells were plated in 96-well plates in 100 μl of media for different days, then each well was pulsed by addition of 10 μl of WST8 and incubated for 3 h. Absorbance readings at a wavelength of 450 nm were taken on Synergy H4 Hybrid Microplate Reader.

### Chromatin immunoprecipitation

786-O/VHL cells were crosslinked with 1% formaldehyde at room temperature for 10 min, and cells were pelleted and resuspended in 400 μl lysis buffer (1% sodium dodecyl sulfate, 10 mM ethylenediaminetetraacetic acid, 50 mM Tris–HCl, pH 8.0). Then DNA of the cells was sonicated and sheared to small fragments of 500–1,000 bp with sonicator ultrasonic processor (Misonix, Farmingdale, NY, USA). Subsequently, the supernatant of the sonicated cells was collected, diluted and precleared by protein A agarose (Santa Cruz Biotechnology). Furthermore, anti-VHL antibody (1:50, Novus biologicals, Littleton, CO) was added to the supernatant for immunoprecipitation with normal preimmuned mouse IgG (1:50, Santa Cruz Biotechnology) as a normal control. After overnight incubation, the protein A agarose were added and incubated for 3 h and then washed with low-salt, high-salt and LiCl buffers and the immunoprecipitated DNA was retrieved by 5 M NaCl at 65 °C for 4 h and purified with a PCR purification kit (TaKaRa). PCR was performed with specific primers for HNF-4α:5′-GGCAGCCTTATCTCTGCAAAAGC-3′ (Promoter, forward) and 5′-GTGGGGGTTAATGGTTAATC-3′ (Promoter, reverse), 5′-GGAGATGACTT GAGGCCTTACT-3′ (3′UTR, forward) and 5′-GGGGAATCGTTTCCAA GGCCTC-3′ (3′UTR, reverse), 5′-GGCTCTGACACTGCAGAGTTCTAGAAC-3′ (Enhancer, forward) and 5′-ACCAACTTACCCAGCTGCTAATCATTGC-3′ (Enhancer, reverse).

### Western blot

Cell extracts were prepared by using the following lysis buffer (4% sodium dodecyl sulfate, 20% glycerol, 100 mM dithiothreitol, Tris–HCl, pH 6.8). In total, 20 μg of proteins were loaded and separated by 10 or 15% sodium dodecyl sulfate-polyacryl-amide gel. After electrophoresis, proteins were transferred to nitrocellulose membrane (Bio-Rad, Richmond, CA, USA). Then, 5% non-fat milk in Tris-buffered saline was used to block the membrane and immunoblotted with antibodies against Flag (1:1,000, Sigma-Aldrich, A8592), Actin (1:10,000, Merck, MAB1501), HIF-1α (1:1,000, BD Transduction Laboratories, 610958), VHL (1:500, Novus biologicals, NB100-485), ALDH2 (1:1,000, Abgent, AM1831a), PDK1 (1:1,000, Stressgen, ADI-KAP-PK112-D) and HNF-4α (1:500, Santa Cruz, sc-6556). Followed by horseradish peroxidase-linked second antibody (1:2,000, Cell signaling Technology, Beverly, MA, USA) for 1 h at room temperature, detection was performed by SuperSignal West Pico Chemiluminescent Substrate kit (Pierce, Rockford, IL, USA) according to the manufacturer’s instructions. All the uncropped versions of images were shown in Supplementary Fig. 10.

### Luciferase assay

The indicated sequences in promoter of HNF-4α were obtained from National Center for Biotechnology Information, amplified by PCR from genomic DNA and subcloned into pGL3-Basic (Promega) to construct luciferase reporter plasmids. For the luciferase assay, 293T cells were seeded in a 12-well plate (Becton Dickinson, Franklin lakes, NJ, USA), and co-transfected with VHL expression vector, luciferase reporter plasmids driven by promoter fragments of HNF-4α and pRLSV40-Renilla. After 36 h transfection, cells were lysed and analysed by the Dual-Luciferase Assay system according to the manufacturer’s instructions (Promega). The following oligonucleotides were used for HNF-4α promoter mutagenesis: the first mutant primers: -104 GGGTCGATGGTGGATCCGTCCCCCGCCGGTGGATAGGCTG -143; the second mutant primers: _-160 ATCCCTGCAGCCATGGCCAGCC TATCCACCG -130; the third mutant primers: -298 GGTGAGTCGACGCACAAAT GAGTGCCCGTGA -268; the forth mutant primers:-423 GCATTGAGGGTAGAA TCTAGAGATTTGGGAAGTTATTG -386; the fifth mutant primers: -419 AATGCTTTTGCAAAGCTTAGGCTGCCCCATGGCCC -453

### *In vivo* studies

Animal care and experiments were performed in strict accordance with the ‘Guide for the Care and Use of Laboratory Animals’ and the ‘Principles for the Utilization and Care of Vertebrate Animals’ and were approved by the Experimental Animal Ethical Committee at Fudan University. Male NOD/SCID or nude mice, 4 to 6 weeks old, were obtained from Shanghai Research Center for Model Organisms. 786-O/EV, 786-O/VHL, Caki-1/NC, Caki-1/sh-VHL or Caki-1/sh-HNF-4α cells were resuspended in matrigel to a final volume of 200 μl and injected subcutaneously. Mice were randomized into vehicle control group or treatment group. When tumours reached an approximate average volume of 50 mm^3^, the mice were treated with 4 mg per kg of doxorubicin. Tumour growth was blinded to measure every 2 days after drug treatment was started. The volume of the tumour was calculated using the equation length × width × width/2.

### Apoptosis assay

Apoptotic cells in the populations were measured with a FACScan flow cytometer (Becton-Dickinson) by the AnnexinV Fluos apoptosis detection kit (Roche Molecular Biochemicals, Mannheim, Germany). Cells were stained with Annexin-V-FITC for exposure of phosphatidylserine on the cell surface as an indicator of apoptosis, following the manufacturer’s instruction (BD Biosciences). Data acquisition and analysis were performed using a BD Biosciences FASCalibur flow cytometer with CellQuest software. Positively stained by annexin-V-FITC only (early apoptosis) and propidiumiodide (late apoptosis) were quantitated, and both subpopulations were considered as overall apoptotic cells.

### Trypsin digestion and stable isotope dimethyl labelling

Cells were dissolved in lysis buffer containing 7 M urea, 2 M thiourea with protease inhibitor cocktail (Roche), followed by centrifugation (15,000 g, 30 min) at 4 °C. The supernatant was quantified using Brandford protein assay, and the precision was validated with SDS-PAGE electrophoresis.

In total, 100 μg of proteins were sequentially reduced with 10 mM DTT and alkylated with 12 mM iodoacetamide. Add trichloroacetic acid to 12% and let it sit for 2 h at room temperature to purify the protein samples. Spin for 5 min in a microfuge (15,000 g) and carefully discharge the supernatant and retain the pellet. Wash the pellet twice with one volume of cold acetone. Vortex and repellet the samples for 5 min at full speed between washes. Samples were reconstituted in 100 μl of 100 mM TEAB buffer. After 1 μg trypsin enzyme was added and digested for 4 h, 2 μg trypsin enzyme was added and incubated overnight. An aliquot of 100 μl of 100 mM TEAB, 8 μl of 4% (vol/vol) formaldehyde (CH_2_O, CD_2_O or 13CD_2_O), 9 μl of 0.6 M cyanoborohydride (NaBH_3_CN or NaBD_3_CN) were added and incubated for 1 h at room temperature. The reaction was quenched with 16 μl of 1% ammonia. Finally, 8 μl formic acid was added to terminate the reaction. The three differentially labelled samples were equally mixed and desalted using SPE-PAK C18(Waters) before LC–MS analysis.

### Two-dimensional LC–MS/MS system

The peptide mixture was fractionated by high pH separation using a UPLC system (Waters Corporation, Milford, MA, USA). Fifteen fractions were collected, each fraction was dried in a vacuum concentrator. The successive low pH separation was achieved with a linear gradient starting from 5% ACN to 40% ACN in 40 min on an EASY-nLC-1200 system combined online with Orbitrap Fusion mass spectrometry (Thermo, San Jose, CA, USA). The mass spectrometer was operated in the data dependent mode collecting full MS scan from 350 to 1,200 *m/z* at 120 K resolution (after accumulation to a target value of 500,000). Ions above the intensity threshold of 2e5 were selected for tandem MS scan at 15 K resolution. Higher energy collisional dissociation (HCD) fragmentation was performed with normalized collision energy of 30.

### Database search and data analysis

The raw data was search against Uniprot human database (2016-3 with 20,211 entries) using MaxQuant (version.1.5.3.7). The light, medium, heavy dimethyl label on both peptide N-terminus and lysine were selected as quantification tags. Peptides with two missed cleavages, fixed carbamidomethylated cysteines and variable acetylated protein N-terminus, and oxidized methionines were set as group-specific parameters. Initial MS and MS/MS tolerances were set at 20 p.p.m. Protein and peptide FDRs were 1%. Quantification results of three biological replications were normalized and the variations were calculated^66^. In each LC–MS run, Maxquant normalize the protein ratios so that the median of their logarithms is zero, which corrects for unequal protein loading, assuming that the majority of proteins show no differential regulation.

Differentially expressed proteins were determined by applying paired *t*-Test with unadjusted significance level *P*<0.05 and corrected by Benjamini–Hochberg algorithm^34^. Multiple test correction was performed by using Scaffold 4 software (version4.7.2, Proteome Software Inc., Portland, OR, USA).

The networks functional analyses were generated through the use of QIAGEN’s Ingenuity Pathway Analysis (IPA, QIAGEN Redwood City).

### Measurement of 4-HNE concentrations

The concentrations of 4-HNE were measured using OxiSelect HNE-His Adduct ELISA Kit (Cell Biolabs) according to the manufacturer’s instructions.

### *C.elegans* experiments

*C. elegans* was maintained on nematode growth medium agar plates seeded with OP50 *Escheria coli* strains at 20 °C. The wild-type strain Bristol N2 and the *vhl-1*-deficient mutant strain CB5602 was obtained from the Caenorhabditis Genetics Center. The double-mutant *acs-20(tm3232);acs-22(tm3236) C.elegans* were obtained from National *BioResource* Project for the Nematode in Japan. RNAi of candidate genes in *C.elegans* was carried out using standard bacterial feeding methods^67^. For RNAi feeding assays, Synchronized L4 worms were placed on each plate seeded with RNAi bacteria and incubated at 20 °C until adulthood, then synchronized for off-spring. The *C.elegans* at L4 stage of the second generation were put into 96-well plates (50–100 per well) with M9 buffer. After treatment with doxorubicin or DMSO for 1–2 days, *C.elegans* deaths were observed with stereo microscope. We used the tip to slightly touch the worms when these worms stop moving, to make sure that these worms are really dead or just stop movements even if they are healthy.

### Statistical analysis

All the statistical analyses were performed by the statistical package for social science (SPSS) (v. 13) (SPSS Institute). The Pearson’s *χ*^2^ test was used to evaluate the correlation among the protein expression of VHL, HNF-4α and ALDH2 in immunochemical assay. Unless described otherwise the *P* values for comparison between line-linked groups were obtained by Student’s two-sided *t*-test. The correlation of relative mRNA levels of VHL, HNF-4α and ALDH2 and its *P* values was analysed using linear correlation and regression. Multigroup comparisons of the means were carried out by one-way analysis of variance test followed by Bonferroni correction for *post hoc* test. *P*<0.05 was considered to be statistically significant.

### Data availability

The authors declare that all the data supporting the findings of this study are available within the article and its Supplementary Information files and from the corresponding authors on reasonable request.

## Additional information

**How to cite this article:** Gao, Y.-H. *et al*. VHL deficiency augments anthracycline sensitivity of clear cell renal cell carcinomas by down-regulating ALDH2. *Nat. Commun.* **8,** 15337 doi: 10.1038/ncomms15337 (2017).

**Publisher’s note:** Springer Nature remains neutral with regard to jurisdictional claims in published maps and institutional affiliations.

## Supplementary Material

Supplementary Information Supplementary Figures

Supplementary Data 1 4282 proteins indentified in RCC4 cells.

Supplementary Data 2 3585 proteins indentified in 786-O cells.

Supplementary Data 3 The dysregulated proteins in RCC4/VHL vs. RCC4/EV.

Supplementary Data 4 The dysregulated proteins in 786-O/VHL vs. 786-O/EV.

Supplementary Data 5 The dysregulated proteins in RCC4/VHL Hypoxia vs. Normoxia.

Supplementary Data 6 The dysregulated proteins in 786-O/VHL Hypoxia vs. Normoxia.

Supplementary Data 7 The proteins regulated by VHL and hypoxia in RCC4 cells.

Supplementary Data 8 The proteins regulated by VHL and hypoxia in 786-O cells.

Supplementary Data 9 The proteins regulated by VHL alone in RCC4 cells.

Supplementary Data 10 The proteins regulated by VHL alone in 786-O cells.

Supplementary Data 11 Comparison of the GO enrichment analysis of RCC4 and 786-O.

Supplementary Data 12 Comparison of the pathway analysis in RCC4 and 786-O.

Supplementary Data 13 The common GO enrichment in RCC4 and 786-O.

Supplementary Data 14 The common pathway in RCC4 and 786-O.

Supplementary Data 15 The potential target genes of HNF-4±.
