# Supplementary material for: VHL deficiency augments anthracycline sensitivity of clear cell renal cell carcinomas by down-regulating ALDH2
Source: Nat Commun. 2017 Jun 15;8:15337. doi: 10.1038/ncomms15337 (PMC5481740; doi:10.1038/ncomms15337)
Supplement: Supplementary Information — Supplementary Figures [file ncomms15337-s1.pdf]

Supplementary figures

a

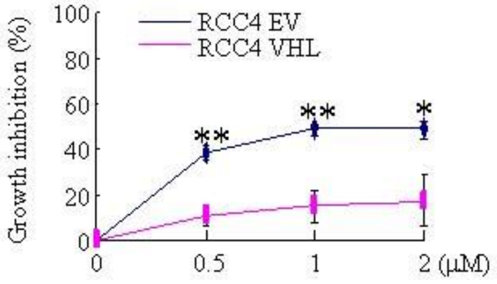

b

	IC50(μM)		
Name	RCC4/EV	RCC4/VHL	Ratio
DNR	0.336	1.752	5.221
EPI	0.726	2.646	3.643
DOX	0.468	1.386	2.962

c

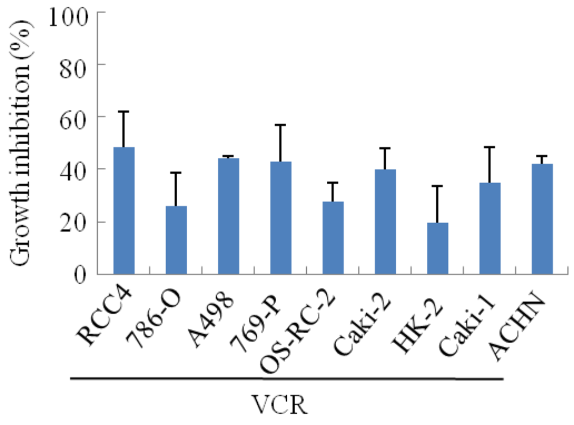

Supplementary Figure 1 | VHL deficiency augments the cytotoxicity of ccRCC induced by anthracyclines. (a) Cell growth inhibition rates of indicated cells treated by indicated drugs with different concentrations for 24 hours. (b) IC50 of RCC4/EV and RCC4/VHL treated with indicated drugs for 48 hours. (c) Different ccRCC cell lines were treated with vincristine for 24 hours and cell growth inhibition rates were detected by CCK-8 and analyzed using ANOVA test followed by Bonferroni correction for post hoc test. The column represents mean with bar as s.d. of three independent experiments with triplicate samples. (*P < 0.05,**P < 0.01 for t-test).

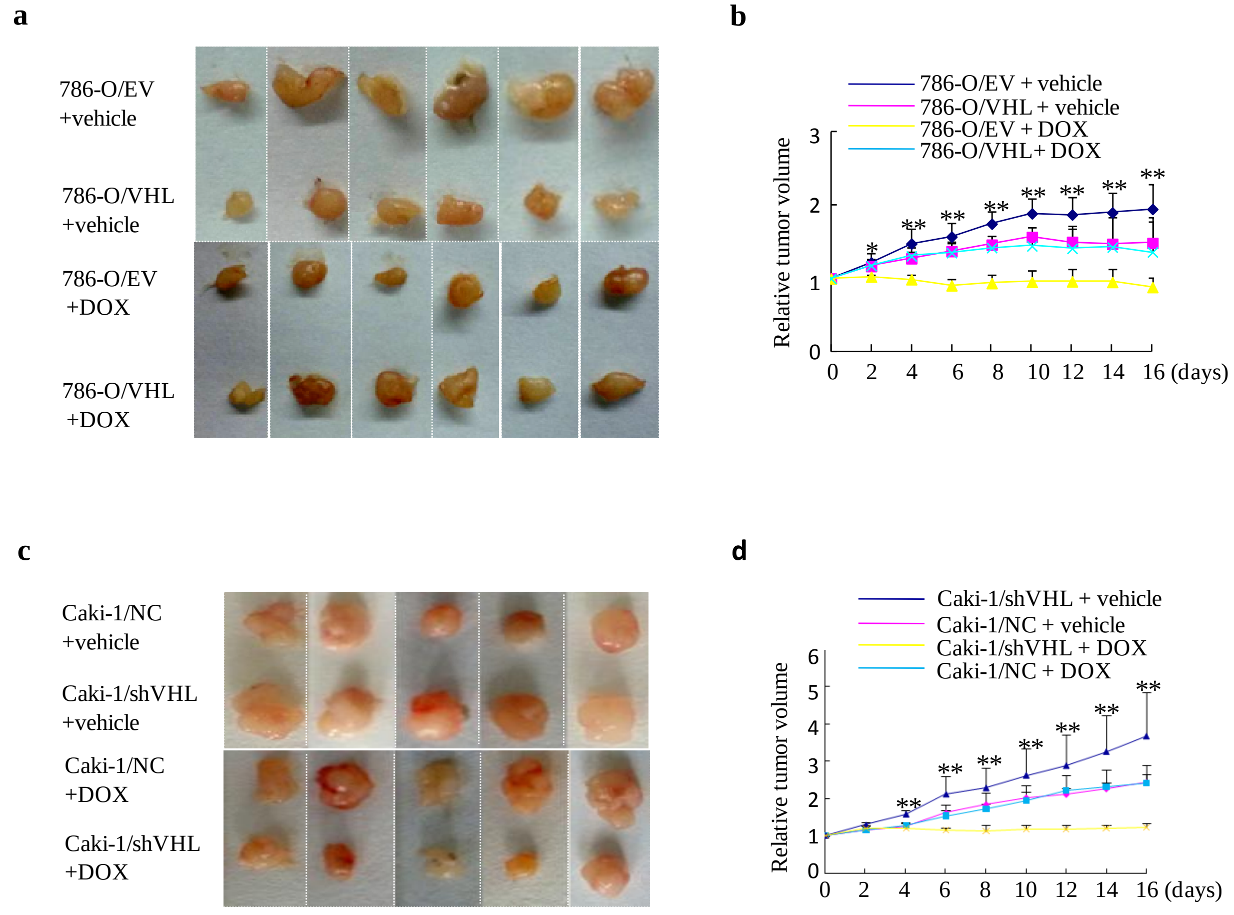

Supplementary Figure 2 | VHL deficiency augments the sensitivity of ccRCC cells to doxorubicin *in vivo*. All indicated cells (5×10^6) were injected into nude mice. Tumor-bearing mice were treated every two days with vehicle or with 4 mg/kg doxorubicin by intraperitoneal injection. At least five tumors per condition were analyzed. (a and c) Representative pictures of tumor in nude mice injected with the indicated cells. (b and d) Relative tumor volume growth. bars, S.D. (*P < 0.05, **P < 0.01 for t-test).

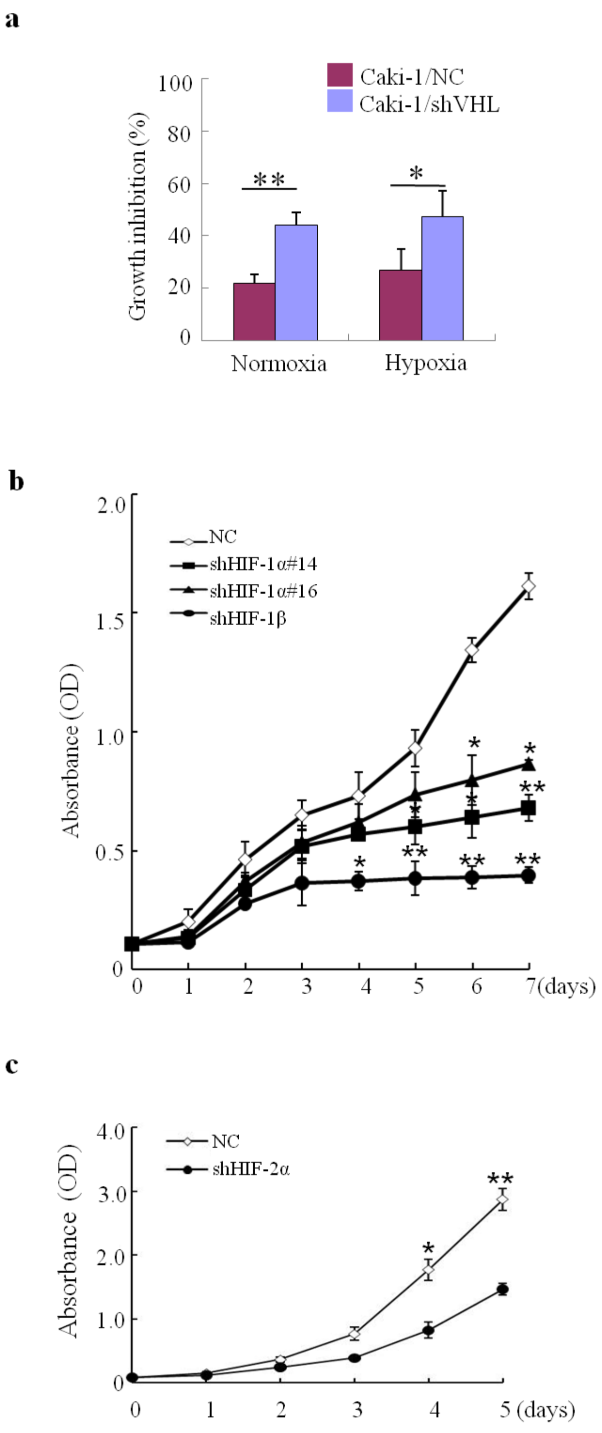

Supplementary Figure 3 | HIF is not involved in doxorubicin-induced cytotoxicity in ccRCC cell lines. (a) Caki-1/NC and Caki-1/shVHL cells were incubated in normoxia and hypoxia for 24 hours, then treated with 1 μM doxorubicin for 24 hours and detected cell growth inhibition rates. (b and c) Growth curves of the indicated cells. bars, S.D. (*P < 0.05, **P < 0.01 for t-test).

a

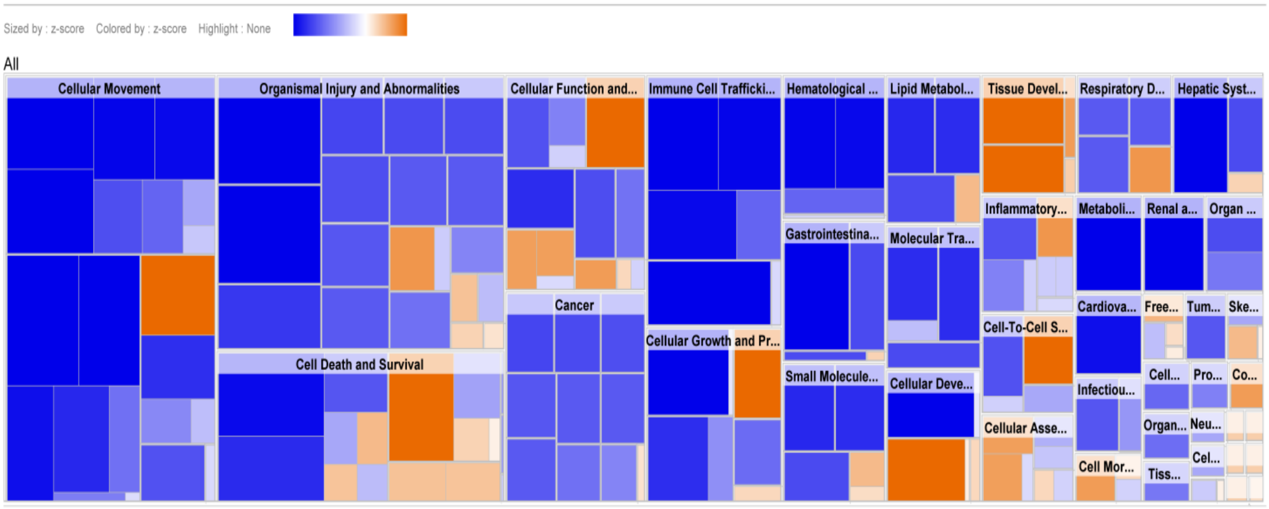

b

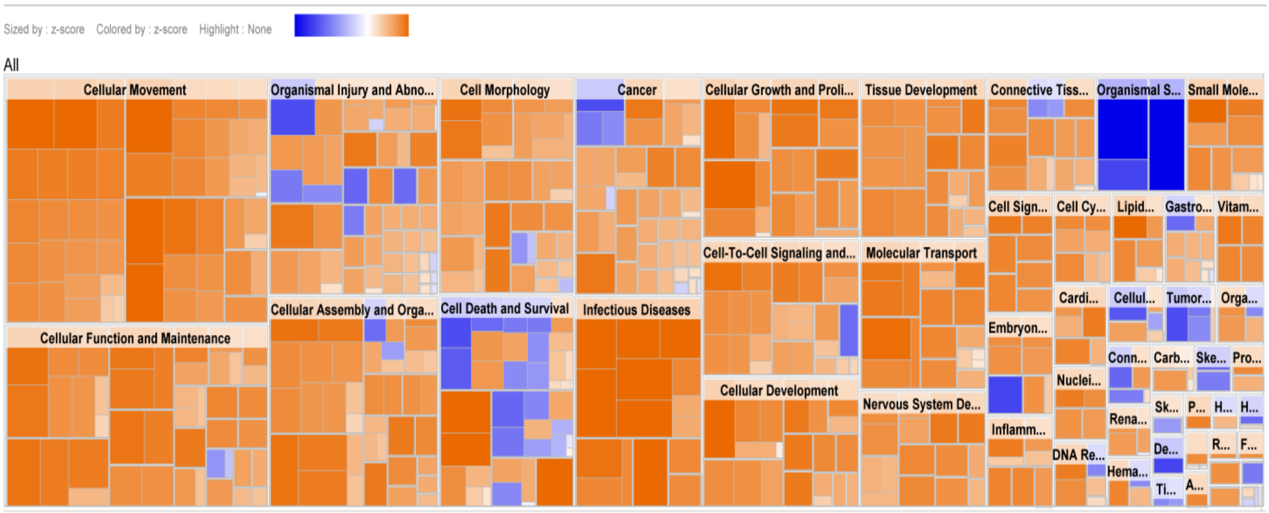

Supplementary Figure 4a,b | Subtractive proteomic analysis reveals candidate proteins regulating cytotoxicity to anthracyclines. Ingenuity Pathway Analysis (IPA) of proteins regulated by VHL alone. The hierarchical heatmap of GO enrichment analysis in RCC4 (a) and 786-O (b). The major boxes represent a category of related functions. Each individual colored rectangle is a particular biological function and the color orange indicates its predicted state: increasing (orange), or decreasing (blue). Darker colors indicate higher absolute Z-scores. In this default view, the size of the rectangles is correlated with increasing overlap significance (using FET *P*-value).

c

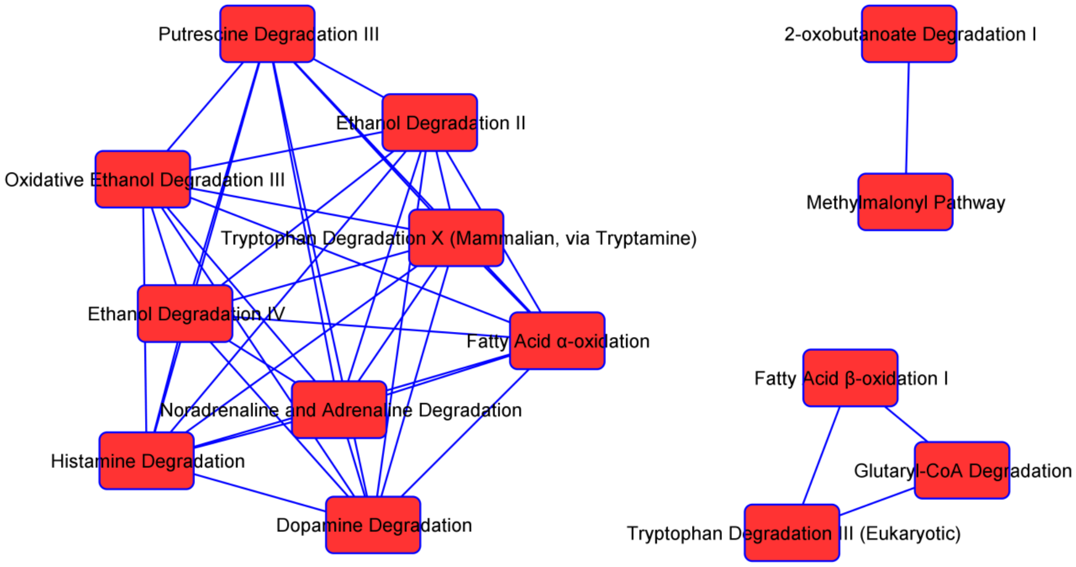

d

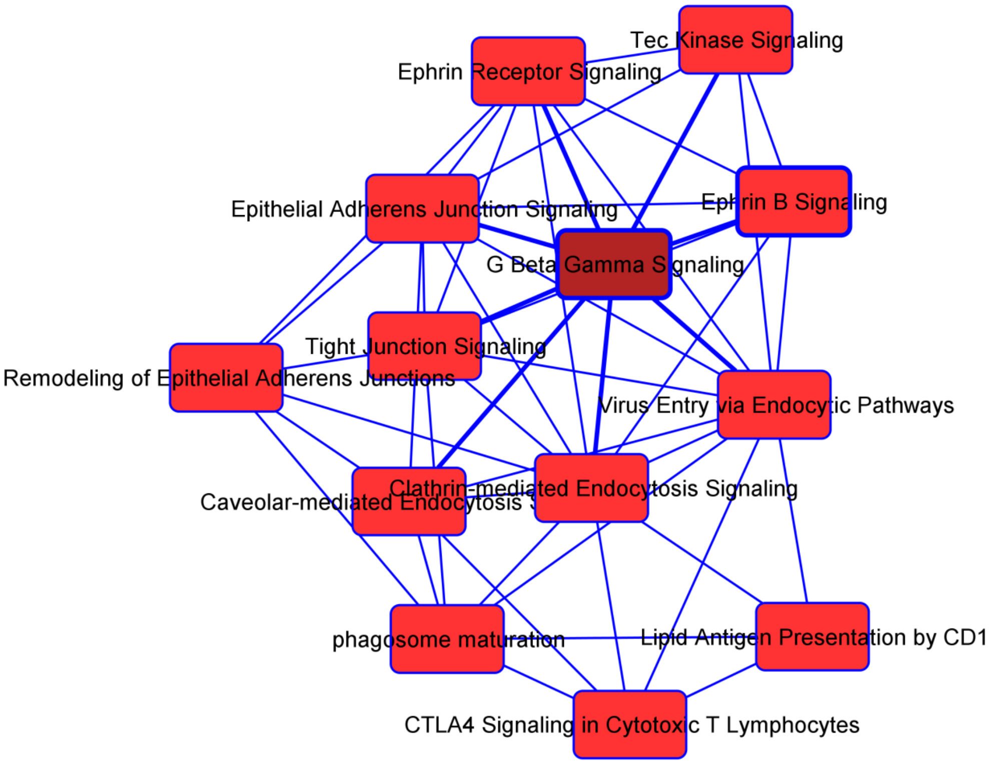

Supplementary Figure 4c,d | Ingenuity Pathway Analysis (IPA) of proteins regulated by VHL alone. The related signaling pathway revealed in the VHL-alone regulated protein in RCC4 (c) and 786-O (d). Each individual colored box represents a particular signaling pathway. The color depth indicates the magnitude of the *P*-value(the deeper the color is, the more significant the signaling pathway). Lines connecting between the rectangles indicate the common proteins involved the signaling pathways.

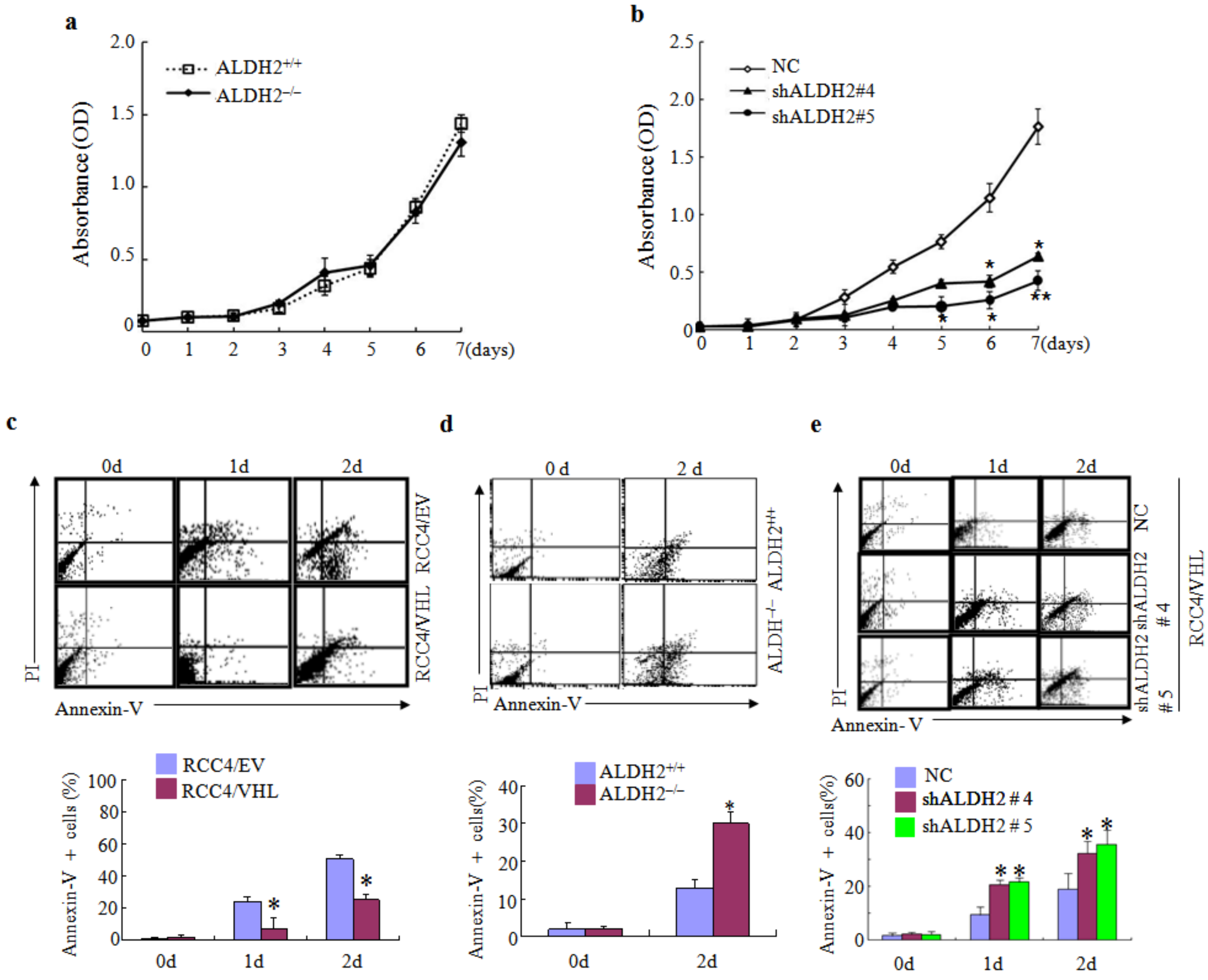

Supplementary Figure 5 | ALDH2 mediates the enhanced cytotoxicity to doxorubicin of VHL-deficient ccRCC cells. (a and b) Growth curves of the indicated cells. (c-e) Cell apoptosis of the indicated cells treated with 1 μM doxorubicin for different time. bars, S.D. (*P < 0.05, **P < 0.01 for t-test).

a

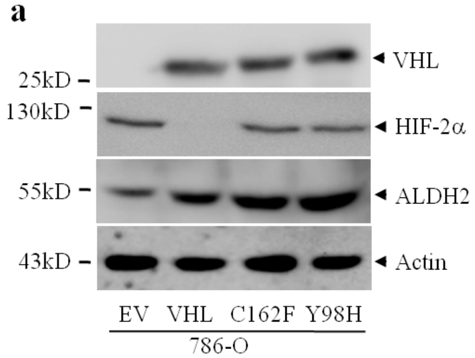

b

Types of mutations	Mutation sites	Mutation domains	E3 ligase activity	No.of patients （n=44）	Ratio
No		No	Yes	31	70.46%
Samesense	L163	α-domain	Yes	4	11.36%
	L188	α-domain	Yes	1	
Missense	H110D	β-domain	No	1	18.18%
	T112H	β-domain	No	1	
	L158V	α-domain	No	1	
	L188M	α-domain	No	1	
	L188Q	α-domain	No	3	
	Q164P	α-domain	No	1	

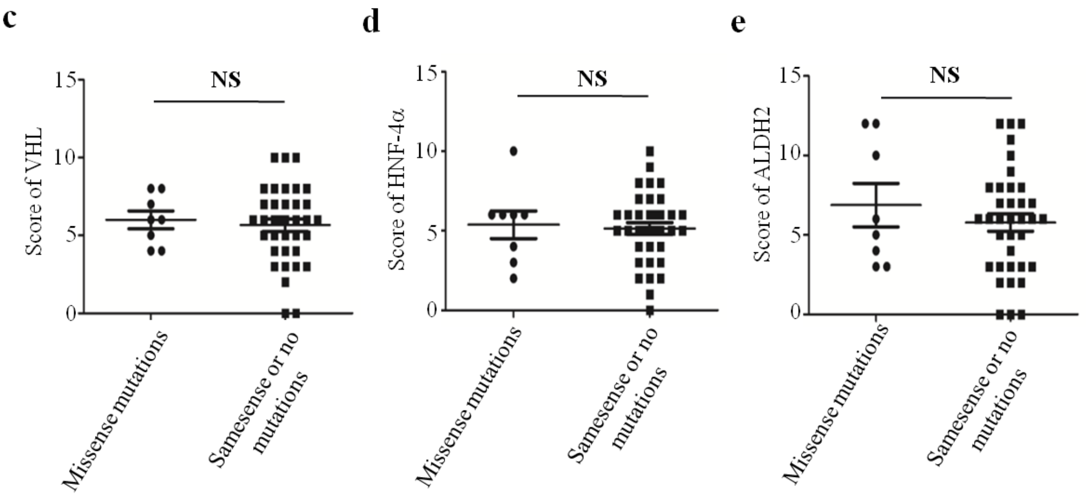

Supplementary Figure 6 | VHL regulates ALDH2 in a HIF-independent manner (a) 786-O cells were stably transfected with VHL expression vector (VHL) or VHL mutation vectors (C162F and Y98H). (b-d) The expression of VHL, HNF-4α and ALDH2 in indicated tissues. Data are shown as mean + s.d. P values were calculated using Student's t-tests. (*P < 0.05, **P < 0.01). NS represents no significant difference.

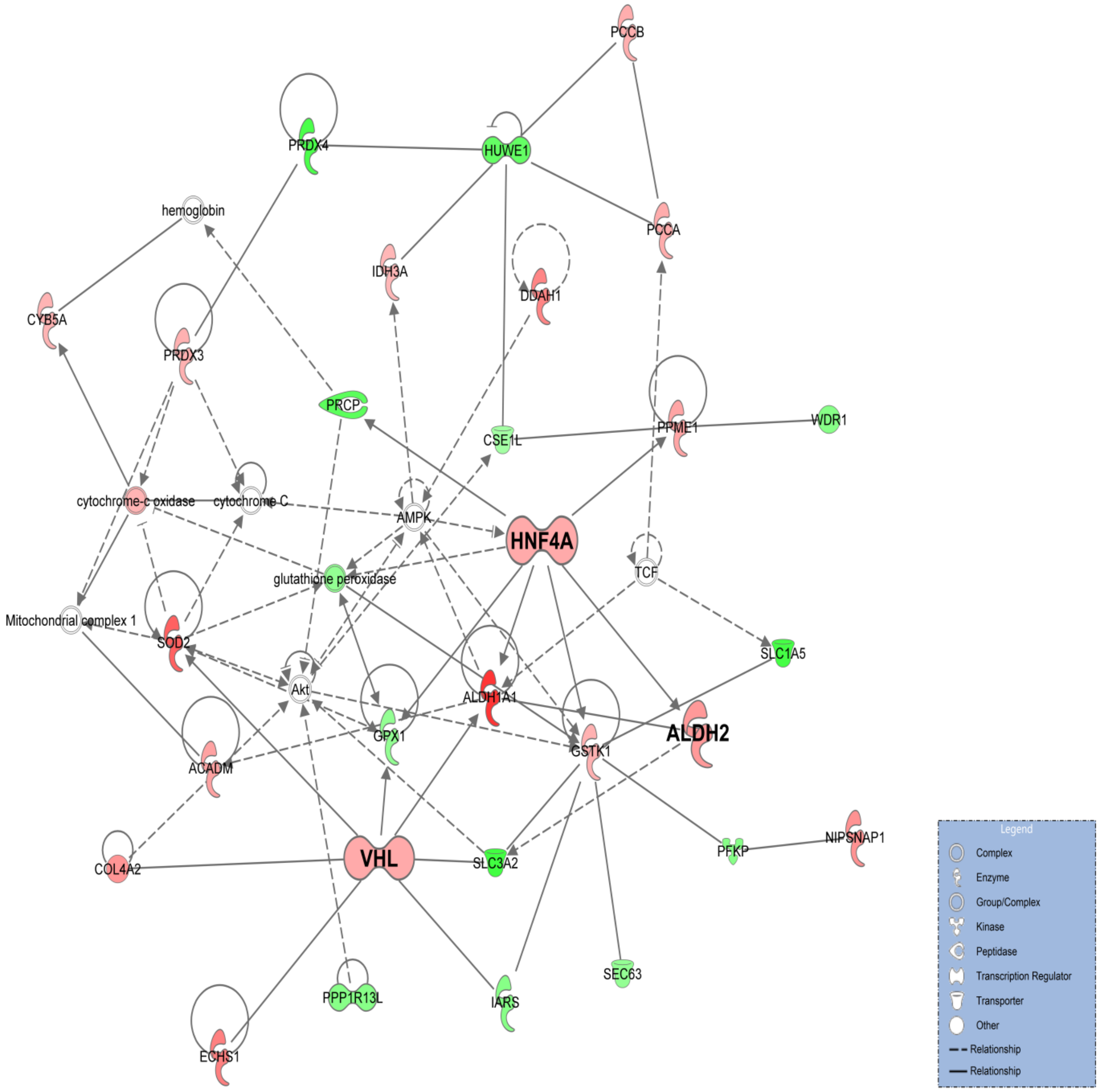

Supplementary Figure 7 | VHL regulates ALDH2 expression by HNF-4α. (a) The network for VHL-alone regulated proteins interacting with VHL, HNF4α and ALDH2 from RCC4 cells. Red, up-regulated proteins; green, down-regulated proteins; and white, proteins known to be in the network but not identified in this study. The color depth indicates the magnitude of the change in protein expression. The shapes are indicative of the molecular class. Lines with arrows connecting between the molecules indicate the molecular relationships. Solid lines indicate direct interactions and dashed lines indicate indirect interactions.

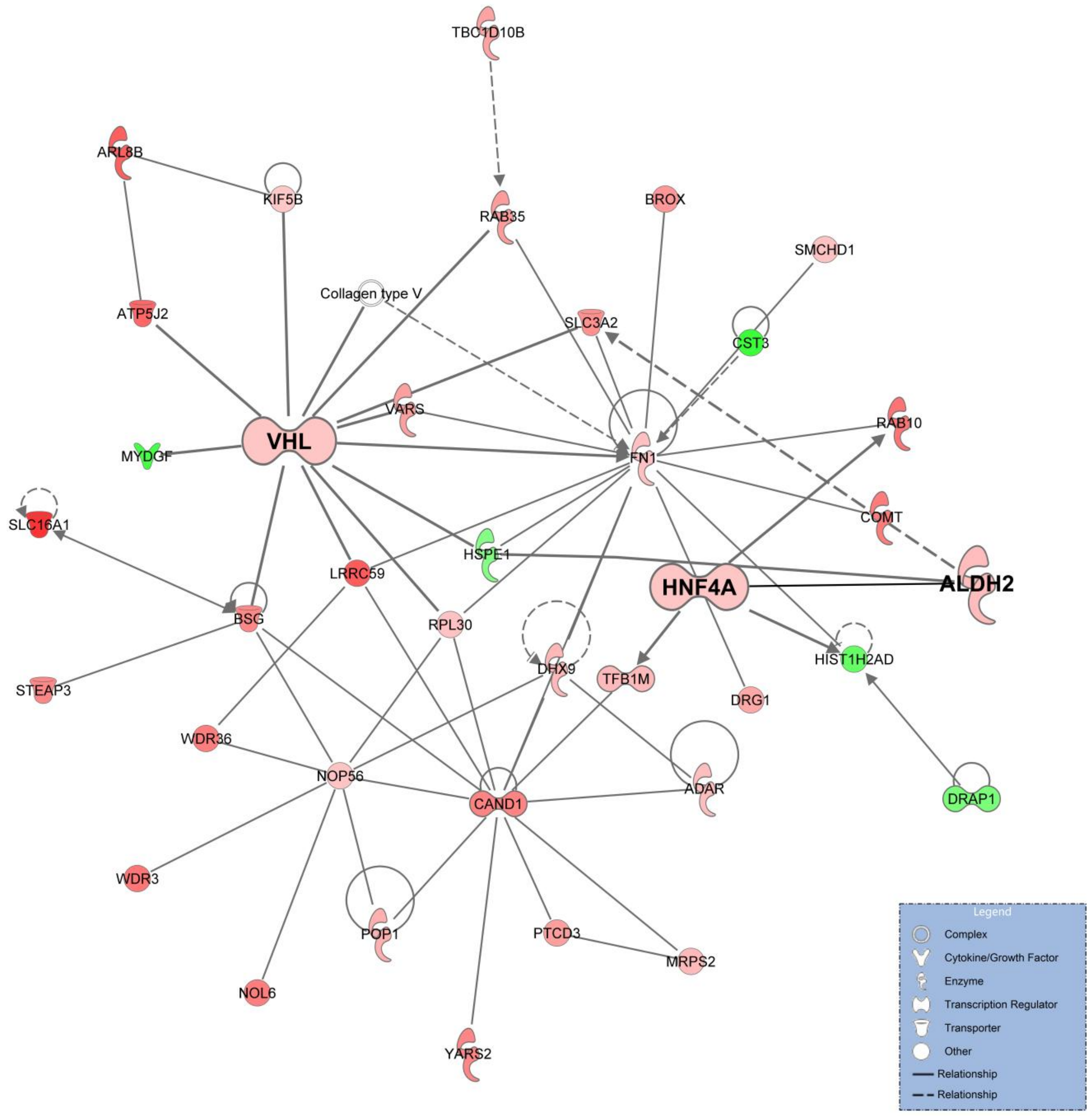

Supplementary Figure 7 | VHL regulates ALDH2 expression by HNF-4α. (b) The network for VHL-alone regulated proteins interacting with VHL, HNF4α and ALDH2 from 786-O cells. Red, up-regulated proteins; green, down-regulated proteins; and white, proteins known to be in the network but not identified in this study. The color depth indicates the magnitude of the change in protein expression. The shapes are indicative of the molecular class. Lines with arrows connecting between the molecules indicate the molecular relationships. Solid lines indicate direct interactions and dashed lines indicate indirect interactions.

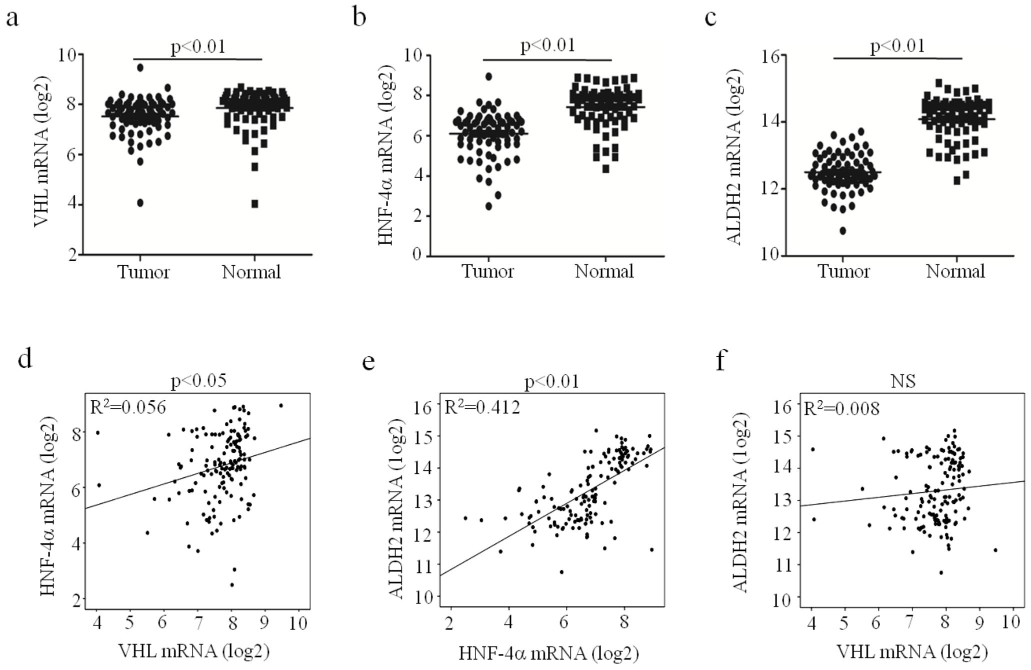

Supplementary Figure 8 | Correlation of VHL, HNF-4α and ALDH2 in ccRCC tissues. (a-c) The mRNA expression of VHL, HNF-4α and ALDH2 in 72 cases of ccRCC patients with tumor and corresponding normal tissues in GEO database (p value is calculated via t-test). (d-f) Correlation analysis of VHL, HNF-4α and ALDH2 mRNA expression (The correlation of relative mRNA levels and the p-values were analyzed using linear correlation). NS represents no significant difference.

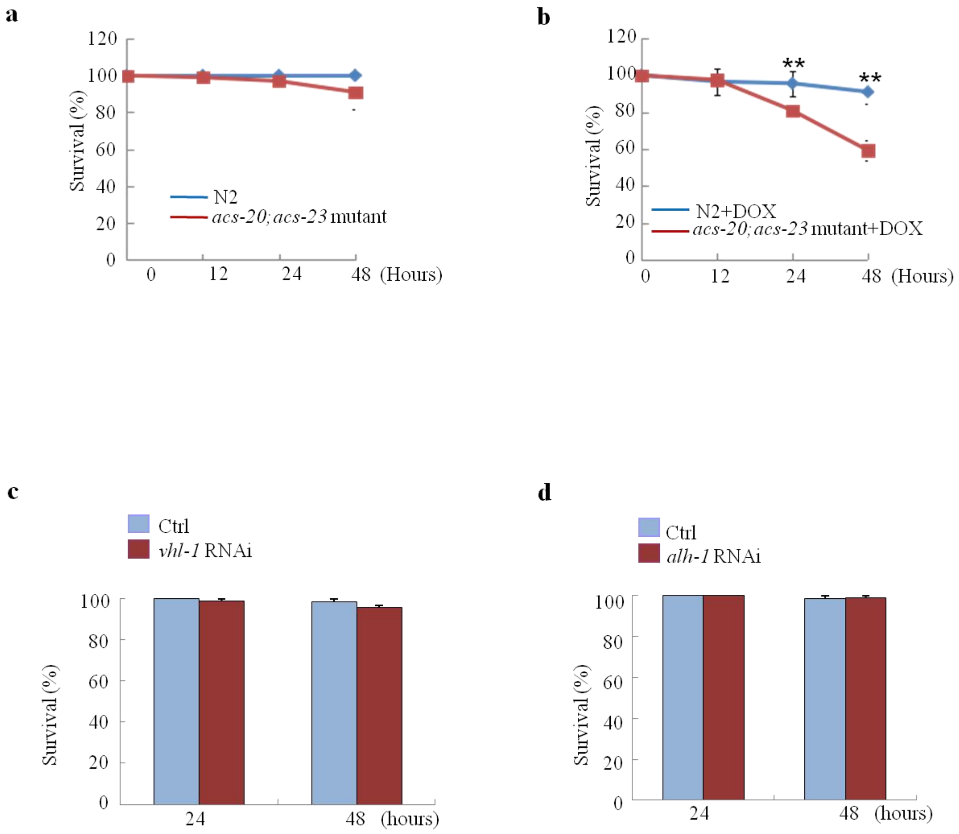

Supplementary Figure 9 | The anthracycline sensitivity and ALDH2 modulation mediated by VHL deficiency is conserved. (a) the effect double mutant of *acs-20;acs-22* on the survival of *C.elegans*. (b) the effect of double mutant of *acs-20;acs-22* on the sensitivity to doxorubicin. (c)The survival of *C.elegans* after *vhl-1* RNAi. (b) The survival of *C.elegans* after *alh-1* RNAi. (*P < 0.05, **P < 0.01 for t-test).

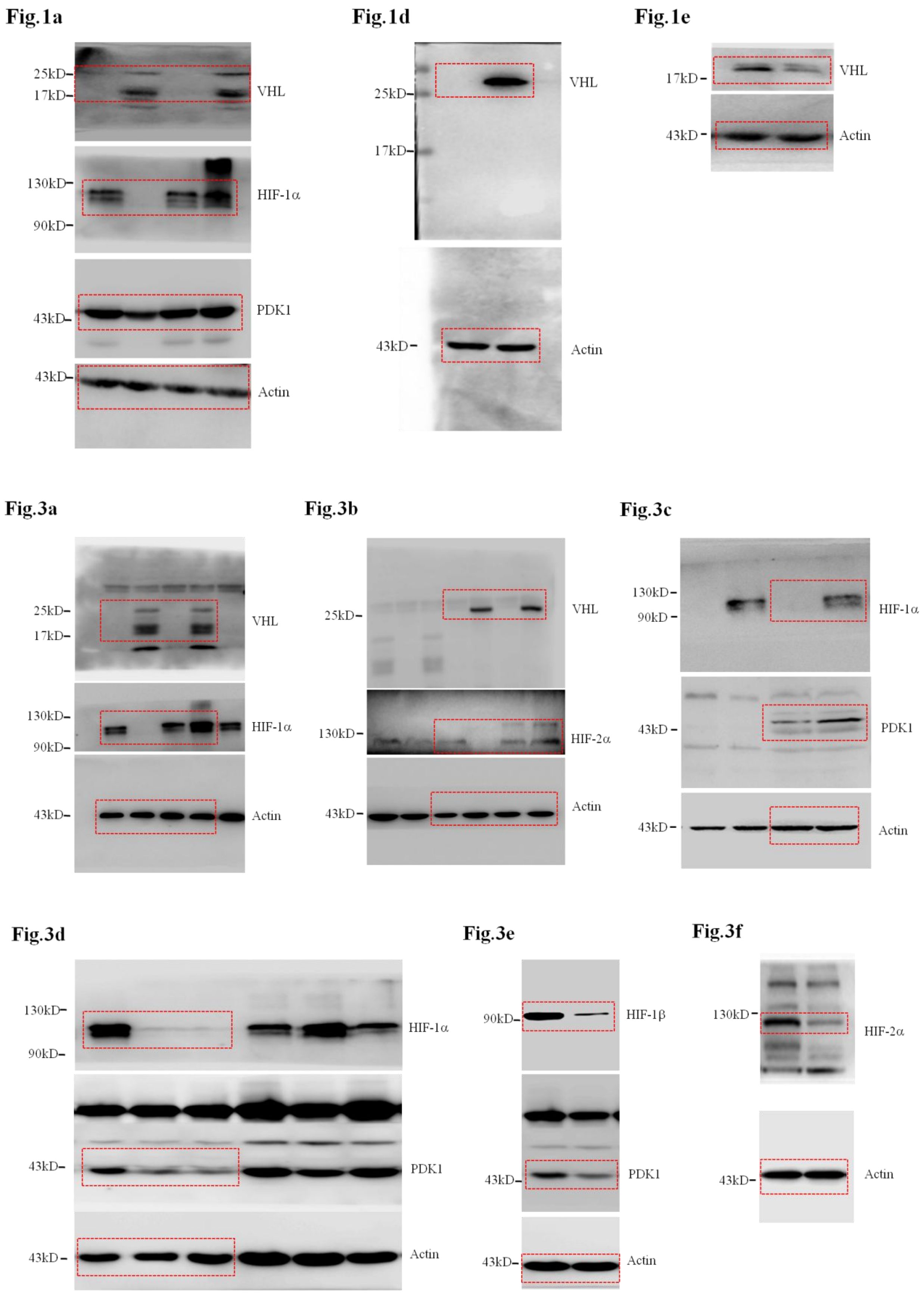

Supplementary Figure 10 | Uncropped gels scans for Figure 1a,d and Figure 3a-3f.

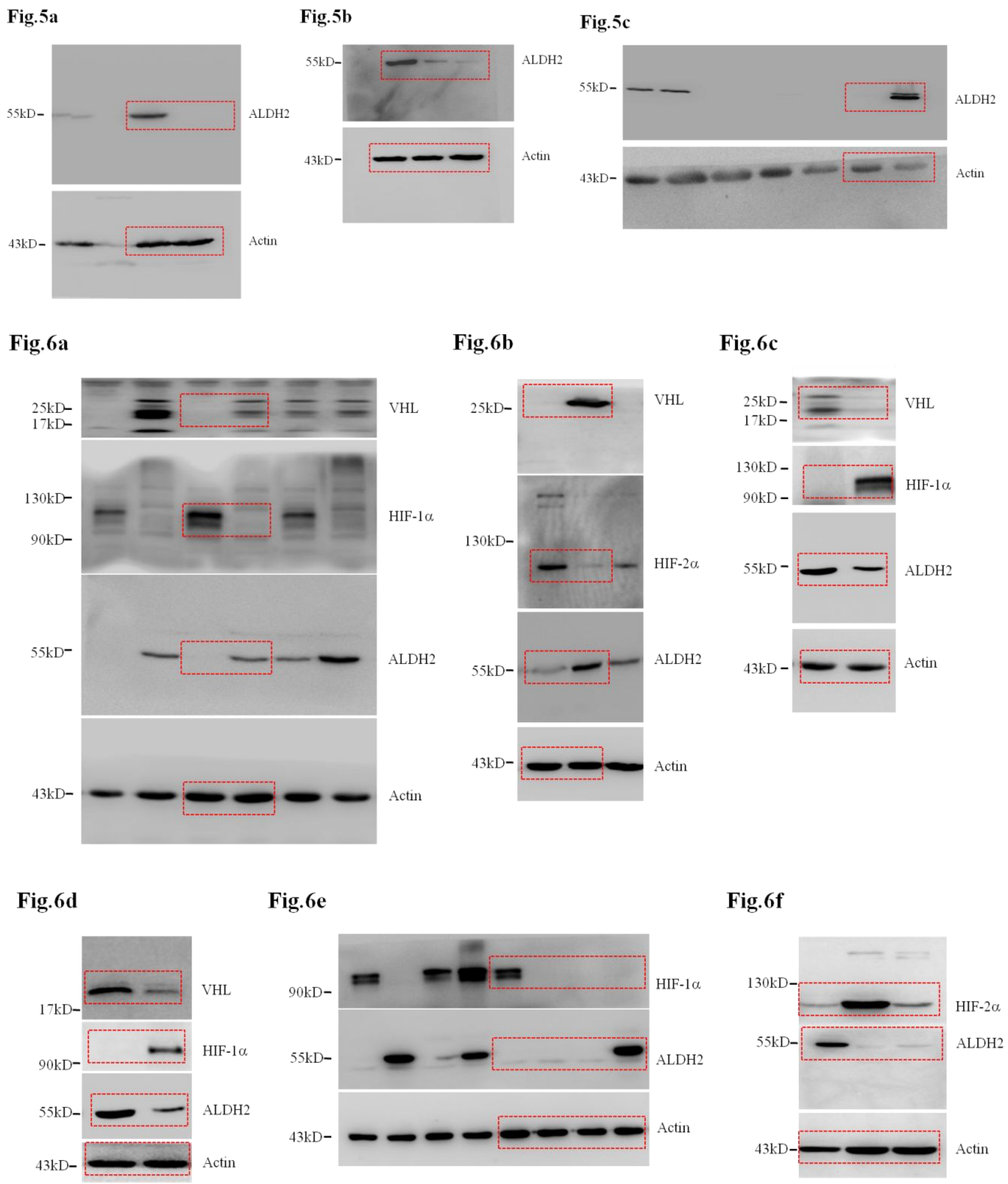

Supplementary Figure 10 | Uncropped gels scans for Figure 5a-5c and Figure 6a-6f.

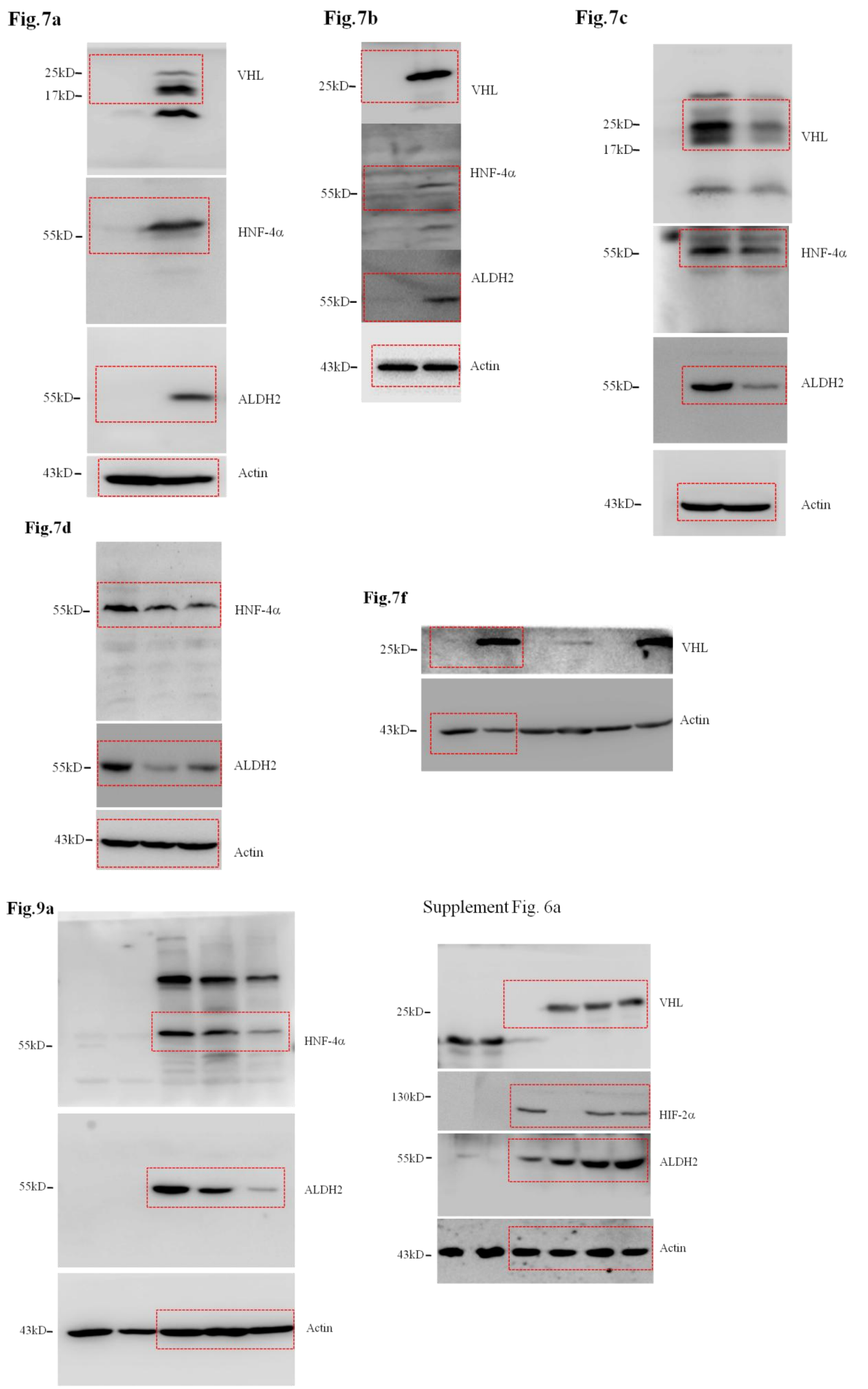

Supplementary Figure 10 | Uncropped gels scans for Figure 7a-7d, 7f, Figure 9a and Supplementary Figure 6a.
